# Latent tuberculosis infection shapes gut microbiome and immune-metabolic interactions in people living with HIV

**DOI:** 10.1080/19490976.2026.2721739

**Published:** 2026-08-27

**Authors:** Pratik Devadiga, Jyoti Batgire, Kalyani Karandikar, Shilpa Bhowmick, Shantanu Birje, Shilpa Kerkar, Vidya Nagar, Priya Patil, Sachee Agrawal, Jayanthi Shastri, Sushma Gaikwad, Kiran Munne, Nupur Mukherjee, Taruna Madan, Vainav Patel, Vikrant M. Bhor

**Affiliations:** a Department of Molecular Immunology and Microbiology, ICMR-NIRWoH, Mumbai, India; b Department of Viral Immunopathogenesis, ICMR-NIRWoH, Mumbai, India; c Department of Child Health Research, ICMR-NIRWoH, Mumbai, India; d Department of General Medicine, Grant Medical College and Sir. J J Hospital, Mumbai, India; e Department of Microbiology, T N Medical College and B Y L Nair Hospital, Mumbai, India; f PI-Centre of Excellence in Immunology, T N Medical College and B Y L Nair Hospital, Mumbai, India; g Department of Medicine, T N Medical College and B Y L Nair Hospital, Mumbai, India; h Department of Cell Physiology and Pathology, ICMR-NIRWoH, Mumbai, India; i Division of Development Research, ICMR, New Delhi, India; j Academy of Scientific and Innovative Research (AcSIR), Ghaziabad, India

**Keywords:** Gut microbiome, HIV, latent tuberculosis, *Prevotella*, predictive functional pathways, short-chain fatty acids

## Abstract

The silent presence of latent tuberculosis infection (LTBI) in people living with HIV (PLHIV) increases the annual risk of tuberculosis (TB) reactivation by 3%–16%. Although gut dysbiosis is well documented in HIV, microbiome alterations associated with LTBI in PLHIV remain poorly characterized. Using integrated cross-sectional and exploratory longitudinal analyses, we investigated the gut microbiome composition, predicted function, and immune-metabolic interactions in ART-naive individuals categorized as HIV−LTBI−, HIV−LTBI+, HIV+LTBI−, HIV+LTBI+, and HIV+TB+. Against the dysbiotic background associated with HIV, LTBI was associated with a distinct gut microbial and immune–metabolic profile. Compared with HIV+LTBI−, HIV+LTBI+ individuals exhibited alpha diversity comparable to the HIV-negative groups while forming a distinct microbial community, characterized by a lower Firmicutes/Bacteroidota ratio, depletion of butyrate-producing Firmicutes, enrichment of ASVs assigned to *Megasphaera*, *Prevotella*, *Bifidobacterium*, and *Streptococcus*, reduced predicted aromatic amino acid metabolic pathways, maintained fecal propionate concentrations, and a densely connected co-occurrence network with greater vulnerability to hub-node loss. Staged correlation analyses revealed progressive remodeling of host‒microbiome associations across HIV and TB disease states, including associations between *Prevotella* ASVs, PD-1+CD8+ T cells, plasma IP-10, and, in HIV+TB+, increased calprotectin and sCD14 together with reduced associations among butyrate-associated taxa. In exploratory longitudinal analyses, ART did not restore a microbiome resembling that of HIV-negative individuals; instead, the microbiome composition remained closer to the baseline HIV+LTBI+ profile, while microbiome divergence and SCFA profiles were associated with the CD4/CD8 ratio rather than CD4 count alone. These findings identify LTBI as an important modifier of the gut microbiome composition and immune-metabolic interactions in PLHIV and provide a framework for future studies investigating microbiome changes associated with progression to active TB.

## Introduction

At the intersection of the syndemic of human immunodeficiency virus (HIV) and tuberculosis (TB), lies the confluence of pathophysiological complexities that remain difficult to fully understand and manage. Globally, an estimated 40.8 million people are living with HIV (PLHIV), including approximately 2.54 million in India, where TB is still endemic. TB exists along a clinical spectrum ranging from latent tuberculosis infection (LTBI), where *Mycobacterium tuberculosis* persists in an asymptomatic state to the active disease characterized by bacterial replication and clinical symptoms.^1^ LTBI brings another layer of vulnerability to the immune system which is already compromised by HIV, with a potential for TB reactivation.^2^ The coexistence of HIV infection and LTBI still remains a critical factor in determining the clinical outcomes and therapeutic strategies despite the transformative impact of antiretroviral therapy (ART).^3^

Gut microbiome, a key non-genetic determinant of host physiology, maintains a bi-directional relationship with the immune system and contributes to overall homeostasis.^4^ Gut microbes can contribute to the disease pathogenesis by exacerbating immune dysfunction, promoting chronic inflammation and compromising mucosal barriers whereas some microbes can also support the immune system and help maintain mucosal barriers by short-chain fatty acid (SCFA) production.^5^ ^,^ ^6^ Individuals infected with both HIV and LTBI have a substantially higher risk of progressing to active TB^2^ and an estimated one-fourth of the Indian population harbors LTBI.^7^ Therefore, it becomes important to understand the changes associated with gut microbiome which can potentially contribute to the eventual reactivation of TB in PLHIV.

In this study, we explored the impact of LTBI on the gut microbiome of individuals with and without HIV. Studying HIV infected ART-naive individuals helps to understand the gut microbiome without the confounding effects of ART-induced immune reconstitution. Comparing microbiome profiles across different stages of HIV and TB coinfection, we identified distinct differences in microbial composition, diversity, functional pathways, metabolites, and co-occurrence networks, specifically in individuals with dual HIV and LTBI infection, differences not observed in HIV-negative individuals by LTBI status. Building on this cross-sectional analysis, we conducted longitudinal follow-up of HIV+LTBI+ individuals after ART initiation to examine how the gut microbiome changes over time and whether it shows signs of recovery toward a healthier state or instead shifts toward a TB-associated profile.

## Materials and methods

### Patient recruitment and clinical assessment

Paired fecal and blood samples were collected at baseline from individuals of age >18 y who were ART naive, HIV-1 positive (*n* = 60) and HIV-1 negative (*n* = 35). At recruitment, a case-record form was used to obtain sociodemographic and clinical variables for all participants, including age, sex, body mass index (BMI), self-reported diet (vegetarian/non-vegetarian), probable route of HIV acquisition/sexual behavior, smoking and alcohol use, and comorbidities (e.g. diabetes mellitus, hypertension, and others) (Table 1). Active tuberculosis along with HIV at the time of recruitment was confirmed by clinical sites for individuals with symptoms by chest X-ray and the GeneXpert test using sputum samples for pulmonary TB and by the FNAC test for extrapulmonary TB. After testing for HIV, all study participants were screened for latent tuberculosis infection (LTBI) based on their clinical history and the IGRA test using the QuantiFERON-TB Gold Plus Kit (Qiagen). Out of 35 HIV-negative individuals, 20 were not latently infected with tuberculosis (HIV−LTBI−) and 15 were latently infected with tuberculosis (HIV−LTBI+). Similarly, out of 60 HIV-positive individuals, 20 were not latently infected with tuberculosis (HIV+LTBI−), 23 were latently infected with tuberculosis (HIV+LTBI+) and 17 were actively infected with tuberculosis (HIV+TB+). The exclusion criteria for the study participants were individuals with autoimmune conditions, pregnancy, antibiotic treatment except for antitubercular drugs for less than 2 weeks and participants with opportunistic infections except tuberculosis. From the HIV+LTBI+ group, 13 participants were followed up prospectively every 6 months for a period of 24 months (Supplementary Table 3). Fecal and blood samples were collected from 13 individuals pre-ART, 13 individuals at 6 months of ART (6M), 12 individuals at 12 months of ART (12M), 8 individuals at 18 months of ART (18M) and 7 individuals at 24 months of ART (24M). 11 out 13 were also started on isoniazid preventive therapy (IPT) around 3 months of ART for a period of 6 months. Two patients were started on cotrimoxazole prophylactic therapy (CPT) along with ART. Participants were recruited from the ART Centre at Grant Medical College & Sir J. J. Group of Hospitals, Mumbai, India (IEC project no: IEC/Pharm/RP/183/Oct/2020) and ICTC and ART Centre of TNMC and BYL Nair Hospital, Mumbai, India (IEC project no: ECARP/2020/152 and ECARP/2020/109) following approval from the National AIDS Control Organization (NACO), Ministry of Health and Family Welfare, Govt. of India and written informed consent. All the samples were processed and analyzed at ICMR-NIRWoH, Mumbai, India (IEC project no:348/2018).

**Table 1.** Demographic and clinical characteristics of the study participants.

LTBI and TB status	HIV-1 seronegative *N* = 35		HIV-1 seropositive (ART naive) *N* = 60			*p*-value
	LTBI -ve (HIV-LTBI−)	LTBI +ve (HIV-LTBI+)	LTBI -ve (HIV+LTBI−)	LTBI +ve (HIV+LTBI+)	Active TB (HIV+TB+)	
Number of subjects	20	15	20	23	17	
Gender (females, males)	12F, 8M	5F, 10M	7F, 13M	8F, 15M	5F, 12M	ns
Females	60%	33%	35%	35%	29%	
Age, median (range)	30 (22–56)	32 (23–48)	37 (23–56)	40 (26–53)	41 (34–59)	0.0056
Diet, *n*	4 Vegetarian; 16 mixed diet	3 Vegetarian; 12 mixed diet	4 Vegetarian; 16 mixed diet	3 Vegetarian; 20 mixed diet	2 Vegetarian; 15 mixed diet	ns
BMI	23.82	25.95	24.32	22.04	19.09	<0.001
CD4 count, median (range)	882 (468–2019)	1012 (552–2478)	442 (50–761)	364 (10–1127)	90 (5–281)	<0.0001
CD4 to CD8 ratio, median (range)	1.68 (0.89–2.54)	1.16 (0.72–2.88)	0.30 (0.02–1.26)	0.39 (0.02–1.56)	0.12 (0.01–0.45)	<0.0001
IFN-γ level on TB1-ag stimulation, median (range)	−0.1 (−2.2 to 0.1)	2.77 (0.29–10)	0.015 (−0.01 to 0.25)	2.34 (0.42–10)	0.22 (−0.02 to 6.55)	<0.0001
IFN-γ level on TB2-ag stimulation, median (range)	0.01 (−3.4 to 0.14)	2.54 (0.18–10)	0.02 (−0.01 to 0.17)	2.23 (0.12–10)	0.32 (−0.02 to 10)	<0.0001
Mode of transmission, *n*	NA	NA	19 Heterosexual; 1 unknown	18 Heterosexual; 1 MSM; 4 unknown	14 Heterosexual; 3 unknown	
Type of TB, *n*	NA	NA	NA	NA	Pulmonary TB (*n* = 10); Extra-pulmonary TB (*n* = 7)	
On TB medication during sample collection, *n*	NA	NA	NA	NA	*n* = 6	
WHO clinical stage, *n*	NA	NA	Stage 1 (*n* = 20)	Stage 1 (*n* = 22); Stage 3 (*n* = 1)	Stage 1 (*n* = 9); Stage 3 (*n* = 3); Stage 4 (*n* = 5)	
Socioeconomic status (Kuppuswamy Scale), *n*	Upper middle (*n* = 18); Lower middle (*n* = 1); Upper lower (*n* = 1)	Upper middle (*n* = 15)	Upper middle(*n* = 1); Lower middle (*n* = 1); Upper lower (*n* = 11); Unknown (*n* = 7)	Lower middle (*n* = 7); Upper lower (*n* = 11); Unknown (*n* = 5)	Lower middle (*n* = 3); Upper lower (*n* = 12); Unknown (*n* = 2)	
Alcohol, *n*	Never (*n* = 13); Occasional (*n* = 5); Habitual (*n* = 2)	Never (*n* = 10); Occasional (*n* = 5)	Never (*n* = 13); Unknown (*n* = 7)	Never (*n* = 13); Occasional (*n* = 1); Habitual (*n* = 3); Unknown (*n* = 6)	Never (*n* = 11); Habitual (*n* = 1); Unknown (*n* = 5)	
Smoking, *n*	Never (*n* = 18); Occasional (*n* = 2)	Never (*n* = 14); Occasional (*n* = 1)	Never (*n* = 13); Unknown (*n* = 7)	Never (*n* = 15); Occasional (*n* = 1); Habitual (*n* = 1); Unknown (*n* = 6)	Never (*n* = 11); Habitual (*n* = 1); Unknown (*n* = 5)	

Participants were stratified according to HIV-1 infection and tuberculosis (TB) status into five groups: HIV-1 seronegative without latent TB infection (HIV−LTBI−, *n* = 20), HIV-1 seronegative with latent TB infection (HIV−LTBI+, *n* = 15), HIV-1 seropositive ART-naive without latent TB infection (HIV+LTBI−, *n* = 20), HIV-1 seropositive ART-naive with latent TB infection (HIV+LTBI+, *n* = 23), and HIV-1 seropositive ART-naive with active TB (HIV+TB+, *n* = 17). Continuous variables are presented as median (range), unless otherwise indicated, and categorical variables are presented as number of subjects (*n*) or percentages. LTBI status was determined using the QuantiFERON-TB Gold Plus (QFT-Plus) assay based on interferon-γ (IFN-γ) responses to TB1 and TB2 antigen stimulation. Active TB was diagnosed using clinical, microbiological, and/or radiological criteria according to standard guidelines. Body mass index (BMI) is expressed as kg/m^2^. CD4 counts are reported as cells/µL. *p*-values indicate comparisons across all five study groups; ns denotes not statistically significant. ART, antiretroviral therapy; LTBI, latent tuberculosis infection; TB, tuberculosis; IFN-γ, interferon-gamma; MSM, men who have sex with men; WHO, World Health Organization; NA, not applicable.

### Sample collection and processing

During participants scheduled visits to the ART centers to collect diagnostic reports or medications, fecal samples, freshly collected at home in sterile containers following standard guidelines, were obtained. The samples were transported to laboratories in cool storage containers, aliquoted and stored at −80 °C for later use. Additionally, during each visit, a 6 ml blood sample was collected in a lithium-heparinized vacutainer for the interferon-gamma release assay (IGRA) using the QuantiFERON®-TB Gold Plus ELISA Kit (Qiagen, Germany). For IGRA, heparinized blood was dispensed into QuantiFERON Nil, TB1, TB2, and Mitogen tubes and incubated at 37 °C for 16–24 h. The harvested plasma after centrifugation was used to quantify IFN-γ as per the manufacturer's protocol. 10 ml blood sample was collected in EDTA vacutainers, out of which 200 µL of whole blood was used for immunophenotyping to determine the absolute counts of activated and exhausted CD4+ and CD8+ T cells subsets. This work is published in our companion study.^8^ The remaining blood was centrifuged at 1400 rpm to separate plasma and was aliquoted and stored at −80 °C until ELISA was performed to study soluble immune markers.

### DNA extraction and 16S rRNA sequencing

DNA was extracted from thawed fecal samples using DNeasy PowerSoil Pro Kit (Qiagen, Germany). The 16S rRNA V3-V4 region^9^ was amplified using the Illumina 16S metagenomic sequencing library preparation protocol. The locus specific primers were 341F (5′-CCTACGGGNGGCWGCAG-3′) and 805R (5′-GACTACHVGGGTATCTAATCC-3′). Each amplification primer carried a 5′ Illumina Nextera overhang adapter—forward 5′-TCGTCGGCAGCGTCAGATGTGTATAAGAGACAG-3′ and reverse 5′-GTCTCGTGGGCTCGGAGATGTGTATAAGAGACAG-3′. Paired-end sequencing of the V3-V4 region of 16S rRNA was performed with 0.5 million reads each in the forward and reverse direction with a read length of 2 × 300 bp using the Illumina MiSeq platform, as described previously.^10^

### Pre-processing of sequencing data and taxonomic assignment

FASTQ files generated after demultiplexing of reads were used to generate amplicon sequence variant (ASV) feature tables using QIIME2 (v2023.2).^11^ The average number of input reads were as follows for cross-sectional group HIV−LTBI− (760,627), HIV−LTBI+ (696,725), HIV+LTBI− (659,112), HIV+LTBI+ (685,670), HIV+TB+ (699,644) and for longitudinal group pre-ART (675,017), 6M (738,110), 12M (739,561), 18M (839,043) and 24M (839,843). Merging of forward and reverse reads, trimming of primers and removal of chimeric reads were done using the DADA2 plugin. Quality assessment was done and reads with a Phred quality score Q of more than 30 were used for subsequent analysis. We obtained a total of 9422 ASVs for the cross-sectional group and 5549 ASVs for the longitudinal samples. Taxonomy-based analyses were performed with a pre-trained Naive Bayes classifier (Greengenes2 2022.10 full-length sequences).^12^ Unassigned ASVs at various hierarchies of taxonomy were reassigned to the rRNA database through NCBI BLAST.^10^

### Diversity analysis

Feature table, taxonomic data, phylogenetic trees generated by QIIME2 and sample metadata were imported into R software and were used to generate a phyloseq object using the phyloseq package (v1.48.0).^13^ Prior to diversity analysis, library sizes ranged from 215,947 to 616,946 reads per sample (median 360,348), and the number of ASVs ranged from 40 to 862 per sample (median 321). To account for differences in sequencing depth, the feature table was rarefied to an even depth of 215,947 reads per sample, which retained all 95 samples. Sampling completeness was verified by per-sample rarefaction curves (Supplementary Figure 2), and Good's coverage which exceeded 99.98% for every sample. Alpha diversity was then characterized using richness estimators (observed ASVs and ACE), diversity indices (Shannon and inverse Simpson), an evenness index (Pielou's evenness, J′) and dominance indices (Berger–Parker dominance, d), which were computed with the vegan package (v2.7-3). Beta diversity was also assessed in R using phyloseq. Distances were computed against a rooted phylogenetic tree using Unweighted UniFrac. Principal Coordinates Analysis (PCoA) was used for ordination (visualized with ggplot2 v3.5.1), and group separation was tested by PERMANOVA (adonis2, vegan) across all pairwise comparisons, with Benjamini–Hochberg correction within each metric (*q* < 0.05). The Venn diagram analysis was done to illustrate the overlap and unique ASVs across different groups. This analysis was conducted using the microeco package (v1.9.1) and visualized with the ggtree package (v3.12.0). The hierarchical clustering tree was visualized using the ggtree package (v3.12.0). ASV counts were used for clustering analysis to generate the dendrogram which visualizes how groups cluster based on overall community composition.

### Bacterial co-occurrence network analysis

Gut microbiome co-occurrence network analysis was performed to assess structural changes in the composition of our study groups. The ggClusterNet (v0.1.0) package was used to perform bacterial co-occurrence network analysis. ASV-level features were retained when present at ≥10 reads in ≥10% of the samples within every group. Pairwise associations between CLR-transformed ASVs were quantified by Spearman rank correlation. An edge was retained when |ρ| ≥ 0.6 and *p*  < 0.05 after Benjamini–Hochberg correction. Key quantitative metrics, including the average degree, which indicates the average number of connections (edges) per node (taxon) within the network, clustering coefficient, which measures the degree to which nodes in a network tend to cluster together and modularity, which measures the structure of the network, indicating how well the network is divided into modules or communities of closely connected taxa, were used to compare network connectivity and community organization across groups. Putative keystone taxa were identified using the within- and among-module connectivity (Zi–Pi) framework.^14^ For every node, the within-module degree z-score (Zi) and the among-module participation coefficient (Pi) were calculated from the module assignment. Nodes were then classified into four topological roles according to established thresholds: network hubs (Zi > 2.5 and Pi > 0.62), module hubs (Zi > 2.5 and Pi ≤ 0.62), connectors (Zi ≤ 2.5 and Pi > 0.62), and peripheral nodes (Zi ≤ 2.5 and Pi ≤ 0.62). Taxa occupying any non-peripheral role were regarded as keystone taxa. Network stability was assessed using robustness to random node loss, robustness to targeted removal of high-degree hub nodes, and overall network vulnerability using the corresponding ggClusterNet package.^15^

### Differential abundance and predictive functional analysis

Differential abundance was assessed with ANCOM-BC2 at the ASV level adjusted for age, BMI, gender and diet. ASVs with ≥10 reads in ≥50% of the samples of at least one group were retained, and each ASV was annotated to the genus level. A global test identified taxa differing across groups, and mdFDR-corrected pairwise post-hoc comparisons (*q* < 0.05) quantified the group-level differences. To study the effect of TB in HIV-positive individuals, only the three HIV-positive groups (HIV+LTBI−, HIV+LTBI+, HIV+TB+) were compared, and the results were visualized as a log-fold-change (LFC) matrix. The comparison across all five groups (HIV−LTBI−, HIV−LTBI+, HIV+LTBI−, HIV+LTBI+, HIV+TB+) was carried out separately, with waterfall plots generated for each pairwise comparison. ASVs significant in the global test were subsequently used for correlation analysis. The Firmicutes/Bacteroidota (F/B) ratio was computed per sample by aggregating ASV counts at the phylum level, summing reads for Firmicutes and Bacteroidota, and taking their ratio with a pseudocount of 0.5 to avoid division by zero. Ratios were log₁₀-transformed prior to analysis. Differences across groups were assessed using the Kruskal–Wallis omnibus test, followed by Dunn's post-hoc pairwise comparisons with Benjamini–Hochberg (BH) correction for multiple testing. The pairwise discriminatory performance of log₁₀(F/B) was then evaluated by receiver operating characteristic (ROC) analysis for every group pair using the pROC package, with the AUC, DeLong standard error, and 95% confidence interval computed for each comparison. The statistical significance of each AUC (versus the null of 0.5) was tested by the Mann–Whitney U test, with *p*-values adjusted across all pairwise comparisons using the Benjamini–Hochberg procedure. Significance was set at adjusted *p* < 0.05. Community functional potential was inferred from the 16S rRNA data using the PICRUSt2 pipeline,^16^ which predicts MetaCyc pathway abundances. The predicted pathways were tested within the same ANCOM-BC2 framework with an abundance ≥1 × 10^−5^ in ≥50% of the samples of at least one group were retained and analyzed in a covariate-adjusted (age, BMI, gender, diet) multi-group model, with global and mdFDR-corrected pairwise comparisons (*q* < 0.05).

### Analysis of fecal and plasma markers for microbial translocation and immune activation

Thawed fecal and plasma samples were assessed for microbial translocation and immune activation markers. 100 mg of fecal sample was diluted with 2.5 ml of PBS. Human secretory immunoglobulin A (sIgA) ELISA kit (Mybiosource, MBS2702203) and Human CALP (Calprotectin) ELISA kit (Mybiosource, MBS7606803) were used to estimate sIgA and calprotectin levels in the diluted fecal samples in accordance with the manufacturer's protocol. The total protein concentration was estimated by Bradford assay and was used to normalize the results. Stored plasma samples were used for estimation of soluble CD14 (sCD14) and retinoic acid by using a Human sCD14 ELISA Kit (Mybiosource, MBS763332) and Human Retinoic Acid ELISA Kit (Mybiosource, MBS705877) in accordance with the manufacturer's protocol. These results were correlated with gut microbiome data.

### Estimation of fecal short-chain fatty acid levels (SCFAs)

Fecal samples were used to estimate SCFAs such as acetic acid, butyric acid, propionic acid, succinic acid and lactic acid by high-performance liquid chromatography (HPLC) by the protocol described earlier.^17^ ^,^ ^18^ Standard curve for each SCFA, expressed in milli-absorbance units (mAU) were established by using known standards, and concentration of unknown SCFAs in fecal samples were determined.

### Correlation analysis of microbiome, immune, metabolite, and cytokine measures across sequential disease states

To capture how associations remodel with disease progression, samples were pooled before correlation into three cohorts, each combining two adjacent groups sharing a broader biological state and defining one transition: HIV−LTBI− to HIV+LTBI−, HIV−LTBI+ to HIV+LTBI+ and HIV+LTBI+ to HIV+TB+. In these states, cross-domain correlation analysis was performed using differentially abundant ASVs (CLR-transformed; Section 2.7), absolute counts of T-cell activation and exhaustion markers, fecal and plasma markers of microbial translocation and immune activation (Section 2.8), and fecal SCFAs (Section 2.9). Within each cohort, pairwise Spearman correlations were computed between features from different measurement sets. Pairs required ≥ 20 paired observations and non-zero variance in both features. *p*-values were adjusted within each cohort using the Benjamini–Hochberg FDR correction *q* < 0.05. All analyses were performed in R.

### Longitudinal gut microbiome analysis

Of the 23 HIV+LTBI+ participants enrolled, only 13 provided consent for longitudinal follow-up stool sampling after ART initiation. Stool samples were scheduled for collection at baseline (pre-ART) and at 6, 12, 18, and 24 months post-ART initiation. The number of participants who provided stool samples at each time point was as follows: baseline (*n* = 13), 6 months (*n* = 13), 12 months (*n* = 12), 18 months (*n* = 8), and 24 months (*n* = 7). ASVs were generated in QIIME2 (2023.2) and analyzed in R using phyloseq, vegan, lme4/lmerTest, ANCOMBC, and MaAsLin2. Low-prevalence features were removed (≥10 reads in ≥20% of samples). Alpha diversity (observed, ACE, Shannon, inverse Simpson, and Pielou's evenness) was computed on rarefied data. On-ART timepoints were contrasted with the pre-ART baseline using linear mixed models with a subject-level random intercept (Benjamini–Hochberg correction), and continuous-time trends were modelled on real elapsed days with random slopes. Beta diversity (unweighted UniFrac) of within-participant dissimilarity from baseline was quantified. To anchor the trajectory clinically, the alpha diversity of the on-ART cohort was compared to that of two cross-sectional comparator groups (HIV-LTBI+ and HIV+TB+) by Kruskal‒Wallis and pairwise Wilcoxon tests, and the mean beta-diversity distances from each on-ART sample to both comparator groups were calculated. Differential abundance versus baseline was tested at the ASV level with both ANCOM-BC2 and MaAsLin2. Because ART was administered to all participants from the 6-month visit onward and was therefore aliased with the timepoint, the models were adjusted only for the covariates that varied within the timepoints, namely, cotrimoxazole prophylaxis (CPT), isoniazid preventive therapy (IPT), and antitubercular therapy (ATT), at the individual level. Finally, microbiome features (alpha metrics and CLR-transformed top ASVs) were correlated (Spearman, BH-corrected) with fecal SCFAs, the CD4/CD8 ratio, and the viral load.

### Statistical analysis

Statistical analysis of differentially abundant taxa, ratios of taxa, alpha diversity, predictive pathway abundance and fecal and plasma markers was performed using the Kruskal–Wallis test followed by post hoc Dunn's test (*p* < 0.05) on GraphPad Prism (v10.1.0). Beta diversity was calculated using Bray–Curtis distance metric to quantify the compositional dissimilarity between sample groups, and PERMANOVA as a non-parametric method to test the significance of group differences based on distance matrices. The results were reported with R^2^, F-statistic, and *p*-value. Receiver operating characteristic (ROC) analysis was carried out using Wilson/Brown method with a 95% confidence interval. Spearman's correlation was used to perform correlation analysis for the gut microbiome data with the ELISA and HPLC data and also for the bacterial co-occurrence networks. The co-occurrence and stability analysis network were filtered for statistical significance (*p* < 0.05) and strong correlations (*r* ≥ 0.6).

## Results

**Figure 1.**
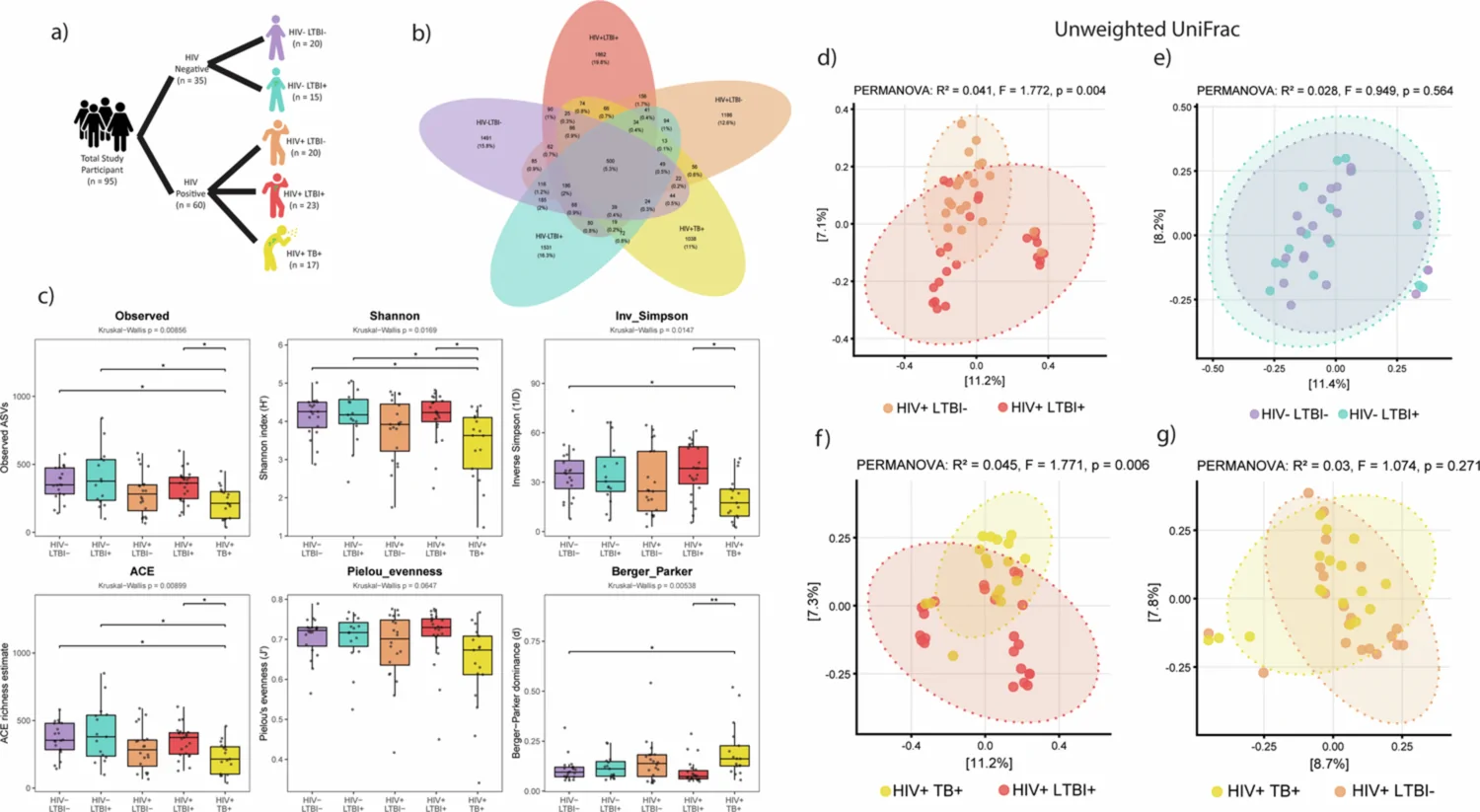
Gut microbiome diversity analysis. (a) Participant recruitment in study groups; HIV−LTBI− (*n* = 20), HIV-LTBI+ (*n* = 15), HIV+LTBI− (*n* = 20), HIV+LTBI+ (*n* = 23) and HIV+TB+ (*n* = 17). All analysis were done with these number of subjects unless specified otherwise; (b) Venn diagram of the distribution of sequencing reads after filtering for Phred score > Q30 in our study groups; (c) alpha diversity estimated by observed features, ACE, Inverse Simpson, Shannon indices, Pielou evenness and Berger Parker; data represented as median (IQR). *p* < 0.05, Significance tested by Kruskal–Wallis test followed by post-hoc Dunn's test; *, *p* < 0.05; (d–g) beta diversity by unweighted UniFrac distance method represented as PCoA plot. Significance tested by PERMANOVA (d) Beta Diversity analysis between HIV+LTBI+ and HIV+LTBI−, F-value = 1.772; R-squared = 0.041; *p*-value = 0.01; (e) beta diversity analysis between HIV−LTBI− and HIV−LTBI+, F-value = 0.949; R-squared = 0.028; *p*-value = 0.602; (f) beta diversity analysis between HIV+LTBI+ and HIV+TB+, F-value = 1.771; R-squared = 0.045; *p*-value = 0.01; (g) beta diversity analysis between HIV+LTBI− and HIV+TB+ F-value = 1.074; R-squared: 0.03; *p*-value = 0.289.

In the cross-sectional investigation, study participants were HIV and TB infected individuals, along with appropriate uninfected controls, and were categorized into 5 groups: (i) HIV−LTBI− (ii) HIV−LTBI+ (iii) HIV+LTBI− (iv) HIV+LTBI+ (v) HIV+TB+ (Figure 1a). ART-naive individuals were recruited to avoid any confounding effects of ART on the gut microbiome composition and diversity at baseline. Groups were designed to allow the following comparisons; (i) HIV−LTBI− vs HIV−LTBI+: to assess the effect of LTBI in the absence of HIV, (ii) HIV−LTBI− vs HIV+LTBI−: to determine changes specifically associated with HIV infection in the absence of LTBI, (iii) HIV−LTBI+ vs HIV+LTBI+: to evaluate the impact of HIV infection in individuals with LTBI, (iv) HIV+LTBI− vs. HIV+LTBI+: to identify key differences associated with the presence of LTBI among PLHIV, (v) HIV+LTBI+ vs. HIV+TB+: to compare latent TB-infected PLHIV with those who have progressed to active TB or have TB reactivation in the context of HIV, and (vi) HIV−LTBI− vs. HIV+TB+: to contrast individuals at the opposite ends of the disease spectrum.

### LTBI is associated with a distinct gut microbiome diversity profile within PLHIV

HIV infection is a well-established driver of gut dysbiosis. We reasoned that if latent or active TB imposes an additional, distinct perturbation on this already disrupted ecosystem, it should be detectable in the overall diversity. We therefore compared alpha and beta diversity across the five study groups. Contrary to the expectation that with an increase in disease severity, the alpha diversity would decrease, alpha diversity was broadly comparable across the HIV-negative groups and the HIV-positive groups without active TB. Kruskal‒Wallis testing indicated overall differences in richness (observed, *p* = 0.0086; ACE, *p* = 0.0090), abundance-weighted diversity (Shannon, *p* = 0.017; Inverse Simpson, *p* = 0.015) and single-taxon dominance (Berger‒Parker, *p* = 0.0054), whereas relative evenness did not differ significantly (Pielou, *p* = 0.065) (Figure 1c). Dunn's post-hoc testing showed that these differences were almost entirely attributable to the HIV+TB+ group, which exhibited significantly reduced richness, diversity and elevated dominance compared to the HIV−LTBI− and HIV+LTBI+ groups (adjusted *p* < 0.05 across Observed, ACE, Shannon, Inverse Simpson and Berger–Parker), as well as compared to HIV−LTBI+ for the richness and Shannon indices. Interestingly, Inverse Simpson exhibited higher diversity in the HIV+LTBI+ group compared to other HIV+ groups, and its median was similar to that of HIV− groups, which suggests that despite coinfection, these individuals maintain a diverse microbiome profile. Because within-sample diversity can be retained even when community members shift, we assessed whether coinfection was associated with differences in between-sample (beta) diversity using Unweighted UniFrac and tested group separation by PERMANOVA with Benjamini‒Hochberg correction (Figure 1d–g). In HIV-negative individuals, LTBI was not associated with any detectable shift in community structure (HIV−LTBI− vs HIV−LTBI+, R^2^ = 0.028, *q* = 0.56). However, HIV+LTBI+ group showed distinct configuration wherein it was significant not only from HIV+LTBI− (R^2^ = 0.041, *q* = 0.01) but also from HIV+TB+ (R^2^ = 0.045; *q* = 0.01), making it the most distinctive community in the cohort. HIV+LTBI− group by contrast, was statistically indistinguishable from HIV+TB+ group (R^2^ = 0.03, *q* = 0.29). Taken together, these findings indicate that, within PLHIV, LTBI is associated with a distinct gut microbial community.

### LTBI in PLHIV is associated with distinct gut microbiome composition

**Figure 2.**
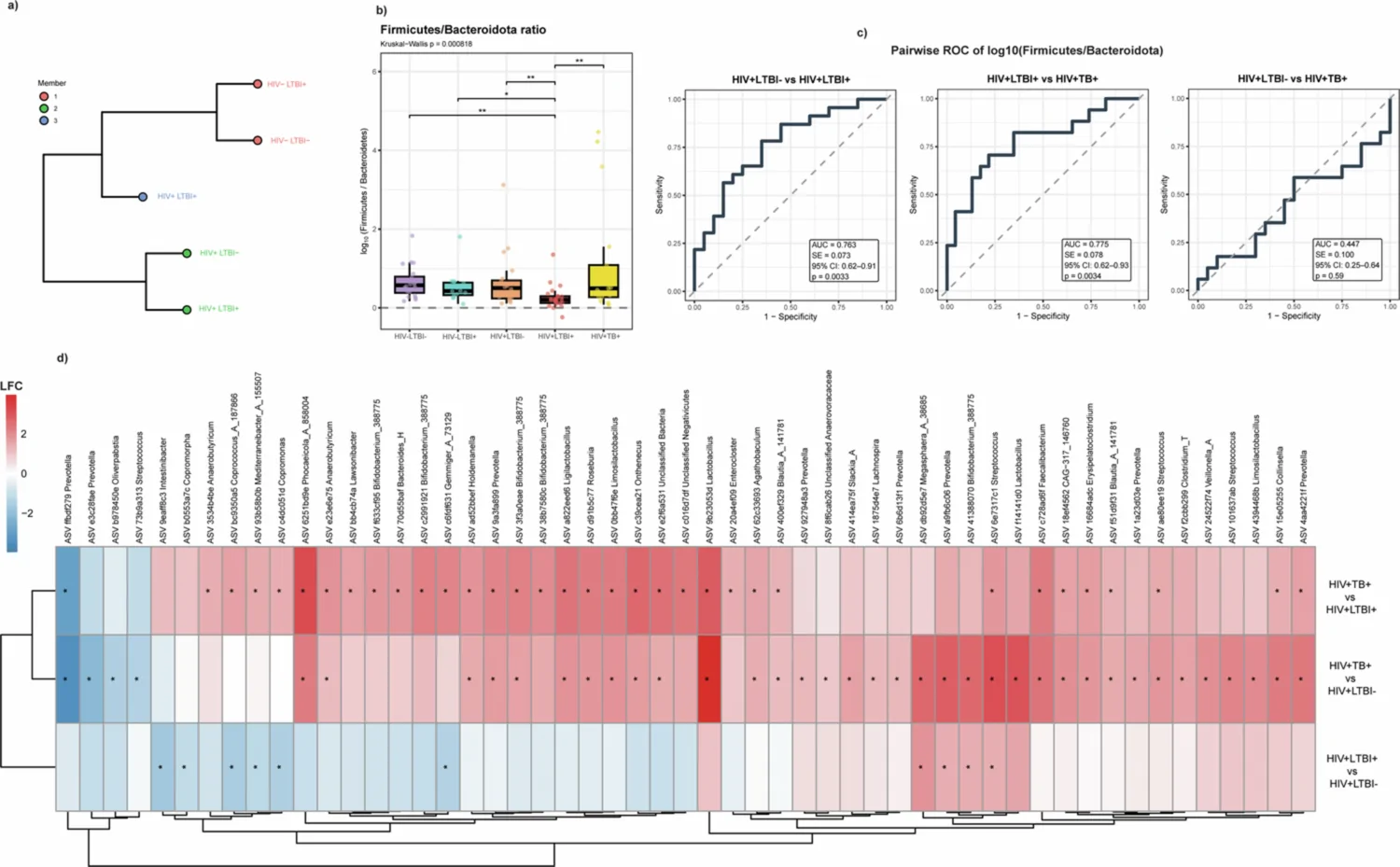
Differential abundance of various taxa in our study group. (a) Hierarchical clustering dendrogram of microbial community profiles aggregated at the study group level. Our five study groups were clustered into three members in a tree denoted by colors red, green and blue; (b) log10 (Firmicutes/Bacteroidota) plotted for the study group; data represented as median (IQR). *p < 0.05; **p < 0.01, Significance tested by Kruskal–Wallis test followed by post-hoc Dunn's test; (c) receiver operating characteristic curve (ROC curve) of log10F/B ratio values to differentiate groups. (d) Heatmap of log-fold-change (LFC) estimates for the amplicon sequence variants (ASVs) found to be differentially abundant in at least one pairwise comparison among people living with HIV and adjusted for age, BMI, gender, and diet. Each column represents one post-hoc pairwise contrast. Cell color encodes the LFC: positive values (red) indicate higher abundance in the first-named group, and negative values (blue) indicate higher abundance in the second-named group. Asterisks (*) mark contrasts that remained significant at a mixed-directional false discovery rate (mdFDR) of *q* < 0.05 under ANCOM-BC2's multiple-comparison correction for the post-hoc (pattern) analysis.

To determine whether HIV and tuberculosis are associated with distinct gut microbial configurations, we first examined the overall community structure. Figure 2a showed the hierarchical clustering dendrogram of gut microbiome showcased HIV−LTBI− and HIV−LTBI+ clustered together, whereas HIV+LTBI− and HIV+TB+ clustered together. Interestingly, HIV+LTBI+ formed a separate third cluster, suggesting that the combination of HIV and latent TB establishes a qualitatively distinct gut ecosystem.

Taxonomic features underlying this separation was consistent with a loss of anaerobic homeostasis characteristic of the HIV associated gut. At the phylum level, Proteobacteria differed significantly across groups (overall *p* < 0.05) and was consistently higher in all the HIV+ groups than in the HIV- groups, especially in HIV+TB+ (Mean relative abundance ± SD; 5.73% ± 8.67) (Supplementary Figure 1). Bacteroidota was also significantly different (*p* < 0.05) and was highest in HIV+LTBI+ (31.78% ± 11.72), being significantly greater than HIV−LTBI− (18.65% ± 10.13), HIV+LTBI− (20.62% ± 11.62) and HIV+TB+ (17.21% ± 12.96), but not the HIV-LTBI+ group (21.83% ± 9.95). This increase occurred at the expense of fermentative Firmicutes, which was significantly lower in HIV+LTBI+ (52.12% ± 10.11) than HIV−LTBI− (65.09% ± 8.50), whereas Actinobacteriota showed no significant difference (*p* = 0.134). The net effect showed a collapse of the Firmicutes/Bacteroidota balance, as shown by (F/B) ratio, which was lowest in HIV+LTBI+ (log_10_ F/B ratio median (IQR); 0.204 (0.123–0.293); overall *p* < 0.05), indicating pronounced dysbiosis in this group (Figure 2b). Because this signature was so specific to HIV+LTBI+, we tested whether the F/B ratio alone could discriminate it from the other groups. Pairwise ROC analysis of the log_10_ F/B ratio discriminated HIV+LTBI+ from every other group (Figure 2c) (HIV+LTBI− vs HIV+LTBI+ AUC = 0.763, *p* < 0.05; HIV+LTBI+ vs HIV+TB+ AUC = 0.775, *p* < 0.05), while comparisons not involving HIV+LTBI+ were non-significant (AUC = 0.48).

To resolve the taxa driving these group differences, differential abundance was assessed at ASV level using ANCOM-BC2, adjusting for age, BMI, gender and diet. Among HIV-positive groups, 75 ASVs were differentially abundant (mdFDR *q* < 0.05) (Figure 2d). Pairwise post-hoc contrasts showed the TB specific signature in HIV-positive individuals was strongly directional, which is consistent with TB reshaping the community. Among the 39 ASVs distinguishing HIV+TB+ from HIV+LTBI−, 35 were enriched in HIV+TB+ and only 4 depleted; of the 34 distinguishing HIV+TB+ from HIV+LTBI+, 33 were enriched in active TB, and only 1 depleted. A core set of 21 ASVs was enriched in HIV+TB+ against both comparators simultaneously (*q* < 0.05), dominated by lactic acid bacteria (*Lactobacillus* ASV 9b23053d, *Ligilactobacillus* ASV a822eed6, *Limosilactobacillus* ASV 0bb47f6e), *Streptococcus* (ASV 6e7317c1 and ASV ae80ee19), *Bifidobacterium_388775* (ASV 3f3a0eae) and SCFA producing Lachnospiraceae/Oscillospiraceae (*Roseburia* ASV d91b5c77, *Faecalibacterium* ASV c728ad6f, *Agathobaculum* ASV 62c33693, *Blautia_A* ASV f51d9f31), *Phocaeicola_A* ASV 6251bd9e and *Collinsella* 15e05255. Although few *Prevotella* ASVs were higher in HIV+TB+, depletion in HIV+TB+ (LFC = <−2) was rare and restricted mainly to two *Prevotella* ASVs (ffbdf279, LFC = −3.47; e3c28fae, −2.22) against HIV+LTBI− and *Prevotella* ASV ffbdf279 (LFC −2.85) was the single taxon lower in HIV+TB+ than HIV+LTBI+. Distinct *Prevotella* ASVs were differentially abundant in opposite directions, suggesting lineage specific rather than genus wide associations. In contrast, 4 ASVs were enriched, and 6 were depleted in HIV+LTBI+ relative to HIV+ LTBI−. Depletion involved a set of commensal Firmicutes, such as *Intestinibacter* ASV 9eaff8c3 (−1.70), *Coprococcus_A_187866* ASV bc9350a5 (−1.58), *Copromonas* ASV c4dc051d (−1.43), *Gemmiger* ASV c6fdf631 (−1.35), *Mediterraneibacter* ASV 93b58b0b (−1.23) and *Copromorpha* ASV b0553a7c (−1.17). This coordinated loss of beneficial fermenters mirrors, at ASV resolution, the phylum-level Firmicutes reduction and low F/B ratio and offers a mechanistic account of why HIV+LTBI+ resolved as its own cluster. Enrichment in HIV+LTBI+ centered on ASVs for taxa such as *Megasphaera* ASV db92d5e7 (LFC = +1.86) *Bifidobacterium_388775* ASV 41388070 (+1.39), *Streptococcus* ASV 6e7317c1 (+1.37) and *Prevotella* ASV a9fb6c06 (+1.36). Finally, in the absence of HIV, HIV−LTBI+ was distinguished from HIV−LTBI− by increased *Prevotella* (ffbdf279, +4.86) and *RUG115* (d3864fc0, +3.32), reinforcing that the pronounced dysbiosis of HIV+LTBI+ emerges specifically from the interaction of HIV and latent TB rather than from either condition alone.

### Distinct microbial network patterns and structural stability in HIV+LTBI+ reveal community disruption and increased susceptibility to perturbation

**Figure 3.**
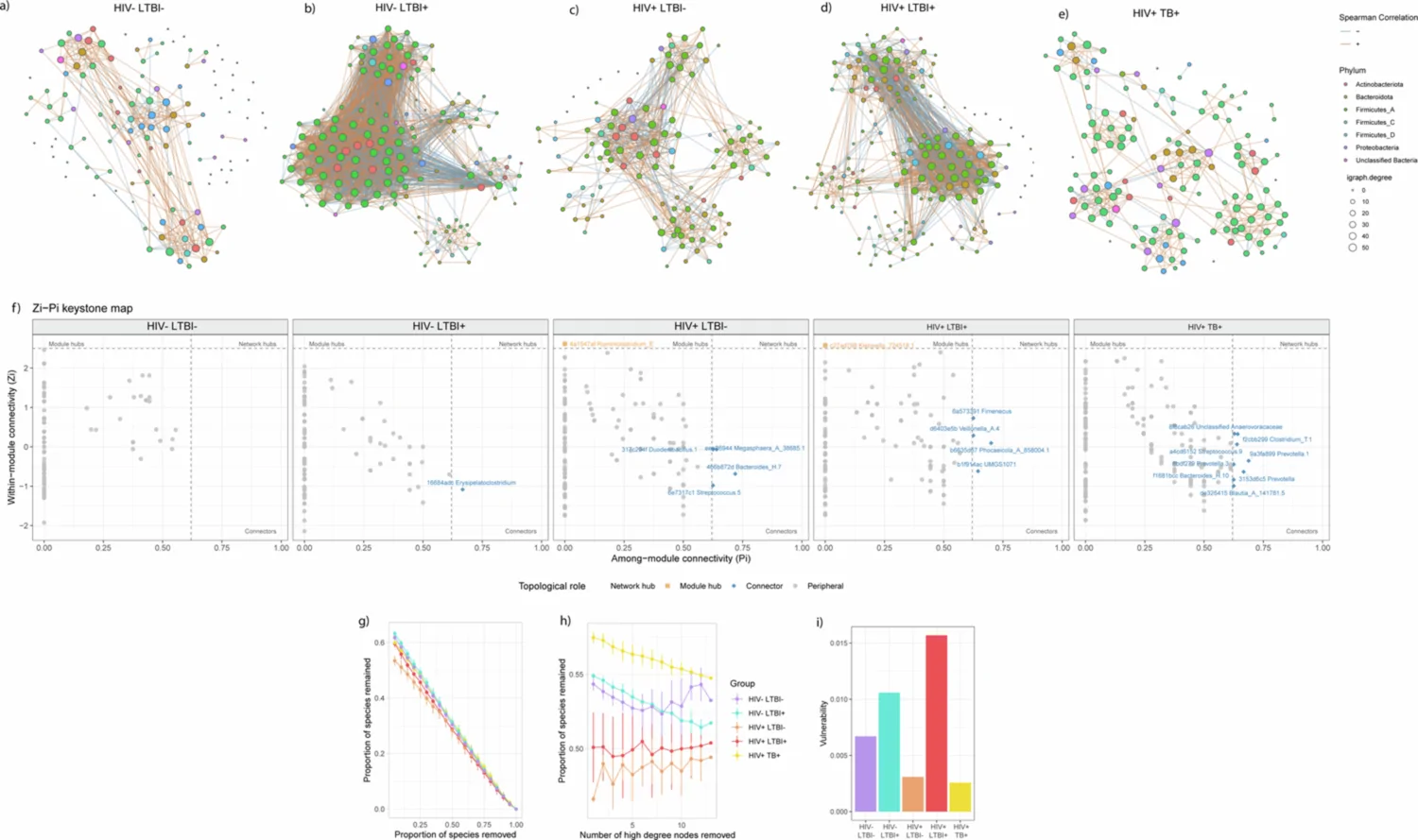
Microbial co-occurrence network analysis: Network graphs were constructed using centered log-ratio (CLR)-transformed ASV abundance data after prevalence filtering (taxa present in ≥10% of samples within each group) and Spearman correlation analysis with Benjamini–Hochberg false discovery rate (FDR) correction (|r| ≥ 0.6, FDR-adjusted *p* < 0.05). Panels (a–e) show the microbial co-occurrence networks for (a) HIV−LTBI− (*n* = 20), (b) HIV−LTBI+ (*n* = 15), (c) HIV+LTBI− (*n* = 20), (d) HIV+LTBI+ (*n* = 23), and (e) HIV+TB+ (*n* = 17). Nodes represent individual ASVs and are colored according to bacterial phylum, while node size is proportional to the igraph degree (number of connections). Edges represent significant microbial associations, with orange and blue lines indicating positive and negative correlations, respectively. (f) Zi–Pi keystone maps showing the topological roles of taxa in each network based on within-module connectivity (Zi) and among-module connectivity (Pi). Taxa were classified as peripheral nodes, connectors, module hubs, or network hubs according to established Zi–Pi thresholds (Zi = 2.5, Pi = 0.62), with labeled taxa representing identified keystone microorganisms. (g) Random node removal robustness curves showing the proportion of taxa remaining as an increasing fraction of nodes is randomly removed from each network. (h) Targeted node removal robustness curves showing the proportion of taxa remaining following sequential removal of the highest-degree nodes, reflecting network resilience to loss of hub taxa. (i) Network vulnerability scores for each study group, where higher values indicate greater susceptibility of the microbial network to perturbation. Robustness and vulnerability analyses were performed on the same CLR-based co-occurrence networks using Spearman correlation (|r| ≥ 0.6, FDR-adjusted *p* < 0.05).

HIV and TB progressively affect the gut microbial composition; therefore, we reasoned that this perturbation would affect the cross talk between the commensals. Co-occurrence network analysis revealed progressive remodeling of potential microbial interactions, wherein ASVs for Bacteroidota, Firmicutes, Proteobacteria and Actinobacteriota shifted markedly in the number and direction of correlation and in their topological roles across the HIV/TB spectrum (Figure 3a–f; Supplementary Table 2). The HIV−LTBI− network was the sparsest and most cooperative of the spectrum (edges = 224; average degree = 4.9; only 18.3% negative edges) and the most compartmentalized, fragmenting into six discrete modules (relative modularity = 0.262). This suggests to be a signature of a healthy gut in which taxa co-vary within niche-specific sub-communities. This compartmentalization collapsed under latent TB as HIV−LTBI+ group produced the densest network (edges = 2399; average degree = 31.4). Among HIV-positive individuals, HIV+LTBI− produced a moderately dense but highly compartmentalized network (edges = 500; degree = 8.9; relative modularity = 0.63), and HIV+TB+ a sparse, elongated and strongly modular network (edges = 309; degree = 4.5; modularity = 0.85; longest path length = 3.75). In contrast to other HIV-positive groups, HIV+LTBI+ carried the most edges (1205) and the highest mean degree (15.2), yet the lowest relative modularity of any group (0.237). Negative edges made up 41.8% of its associations, close to HIV−LTBI+ group (43.7%) and far above HIV+TB+ (13.3%), pointing to competition-driven rather than cooperative co-abundance. Notably, ASVs for *Klebsiella* rose to the module-hub status here, which is the only Proteobacterial pathobiont to anchor any group's network alongside connector keystone ASVs for *Phocaeicola_A*, *UMGS1071*, *Veillonella_A* and *Fimenecus*. HIV+LTBI+ was also the most vulnerable network on the spectrum (0.0157), five-fold HIV+LTBI− (0.0031) and six-fold HIV+TB+ (0.0026), exceeding even latent TB (0.0106) (Figure 3i). Robustness to targeted highest-degree nodes removal was correspondingly lower in the HIV-positive than the HIV-negative networks, whereas random node loss degraded all groups near-identically (slopes ≈ −0.6)—localizing the fragility of HIV+LTBI+ to its dependence on a few influential nodes rather than to random attrition.

### Metabolic functional pathway shifts in gut microbiome are associated with HIV and TB coinfection

**Figure 4.**
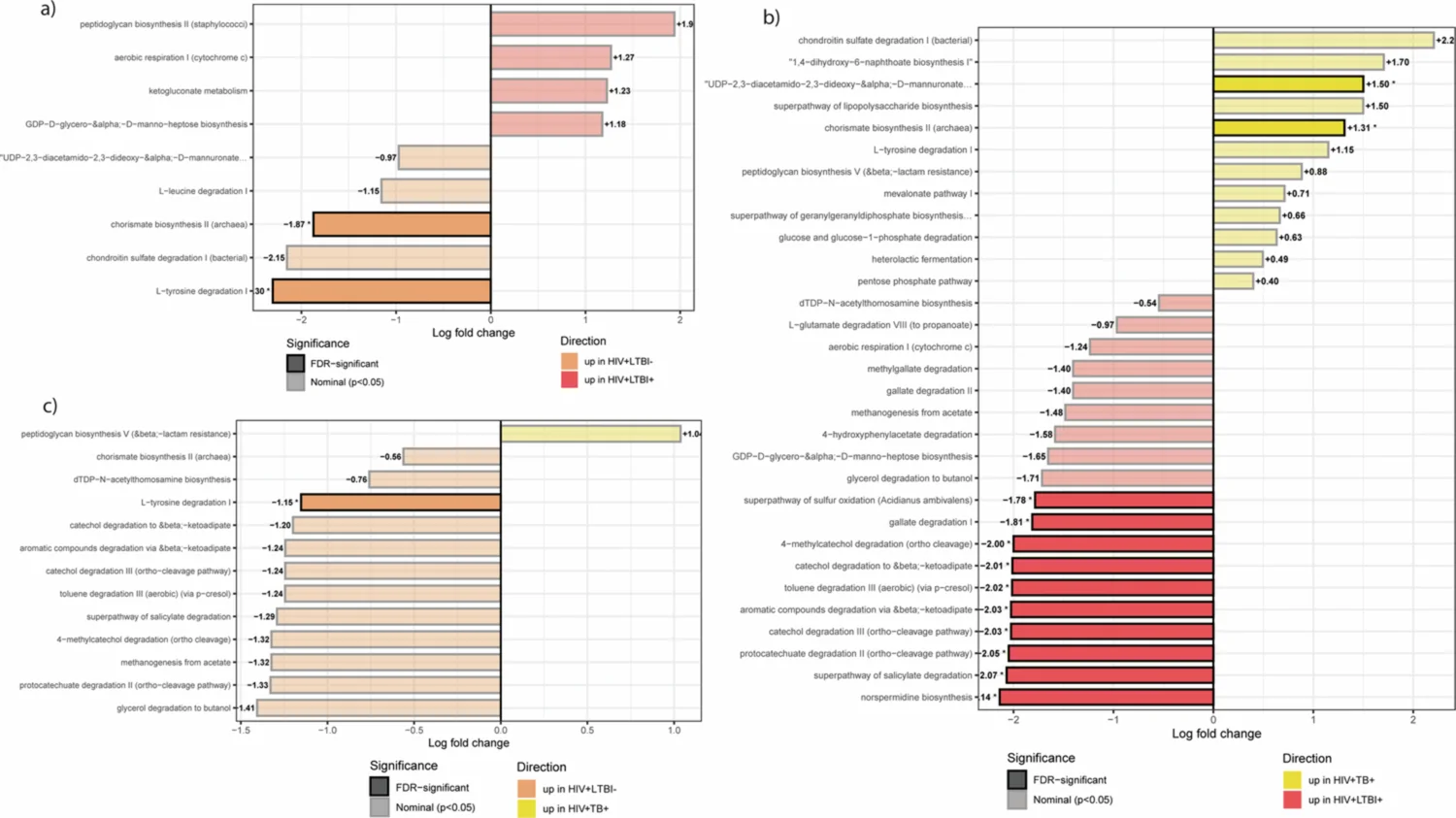
Differentially abundant PICRUSt2-predicted metabolic pathways identified by ANCOM-BC2 pairwise comparisons. Waterfall plots showing pathways with nominal significance (*p* < 0.05), with pathways remaining significant after mdFDR correction (*q* < 0.05) indicated by an asterisk (*). Bar length represents the log fold change (LFC), where positive values indicate enrichment in the group indicated by the darker color in the legend, and negative values indicate enrichment in the comparison group. Dark-colored bars denote FDR-significant pathways, whereas lighter-colored bars represent pathways that were significant only at the nominal level (*p* < 0.05). (a) HIV+LTBI+ versus HIV+LTBI− comparison. (b) HIV+TB+ versus HIV+LTBI+ comparison. (c) HIV+TB+ versus HIV+LTBI− comparison.

The change in composition of gut microbiome associated with HIV-TB coinfection, whether also reshapes its functional potential, was studied based on the multi-group ANCOM-BC2 analysis of PICRUSt2 inferred MetaCyc pathways. After covariate adjustment and mdFDR correction, HIV−LTBI+ differed from HIV−LTBI− in just one decreased predicted pathway (decaprenyl phosphate biosynthesis; *q* = 0.013), suggesting that latent TB has a negligible functional imprint on the HIV-negative individuals gut microbiome. In sharp contrast, HIV+LTBI− group showed an increase in 49 pathways compared to HIV−LTBI− (q < 0.05), dominated by an increase in respiratory-quinone biosynthesis (menaquinone/demethylmenaquinone superpathways, 1,4-dihydroxy-2-naphthoate and phylloquinol routes), heme biosynthesis, glyoxylate-bypass and 2-methylcitrate central-carbon metabolism, enterobactin-type iron acquisition, and (Kdo)₂-lipid A/LPS, enterobacterial-common-antigen and polymyxin-resistance envelope functions. Collectively, the predicted signature of a facultatively anaerobic, respiring, iron-scavenging Gram-negative (Enterobacteriaceae/Proteobacteria) expansion, the functional counterpart of mucosal barrier injury and microbial translocation characterizes HIV enteropathy. Similarly, in HIV+LTBI+ group, 63 pathways were differentially abundant from HIV−LTBI+ and 59 pathways from HIV−LTBI− (*q* < 0.05). 45 of those pathways enriched in HIV+LTBI+ relative to HIV−LTBI− were the same pathways enriched in HIV+LTBI− relative to HIV-LTBI−, reproducing identical respiratory-quinone, heme, glyoxylate/methylcitrate, siderophore and LPS related pathways. Yet, HIV + LTBI+ when compared with HIV+LTBI− resulted in only two pathways. L-tyrosine degradation I (LFC = −2.30, *q* < 0.05) and chorismate biosynthesis II (LFC = −1.87, *q* < 0.05) were each predicted to be lower in HIV+LTBI+ than in HIV+LTBI−. L-tyrosine degradation I was indistinguishable between HIV+LTBI− and HIV−LTBI−, but was lowest specifically in HIV+LTBI+ and recovered only partially in HIV+TB+ (HIV+TB+ versus HIV+LTBI+, LFC = +1.15, nominal *p* < 0.05). The single pathway that distinguished HIV+TB+ from HIV+LTBI− was again L-tyrosine degradation I (LFC = −1.15, *q* = 0.037). The predicted tyrosine catabolic capacity of the gut microbiome is thus highest in HIV alone and is suppressed whenever a HIV and TB coinfection takes place, especially in latent TB infection. Chorismate biosynthesis II pathway also showed similar complementary trend, wherein, HIV+LTBI− relative to HIV-LTBI− (LFC = +2.23, q < 0.05), returned to the baseline in HIV + LTBI+ (HIV+LTBI+ versus HIV−LTBI− was non-significant; versus HIV+LTBI−, LFC = −1.87), and again increased in HIV+TB+ (HIV+TB+ versus HIV+LTBI+, LFC = +1.31, *q* < 0.05). Importantly, the broader superpathway of chorismate metabolism was uniformly and significantly elevated across all three HIV-positive groups relative to both HIV-negative groups (HIV+LTBI−, HIV+LTBI+ and HIV+TB+ all *q* < 0.05, with no differences among them). Consistent with this, 12 pathways separated HIV+TB+ from HIV+LTBI+ group. A broad range of aromatic-ring degradation pathways (protocatechuate and catechol ortho-cleavage, gallate, salicylate and toluene degradation, and β-ketoadipate funnel), together with norspermidine biosynthesis and a sulfur-oxidation superpathway, was predicted to be higher in HIV+LTBI+ and to reduce with HIV+TB+, while chorismate biosynthesis II and UDP−2,3-diacetamido-2,3-dideoxy-α-D-mannuronate biosynthesis (a Gram-negative envelope sugar) increased in HIV+TB+ group. Together, these predicted shifts indicate that the gut microbiome's functional contribution associated with HIV-TB co-infection is overwhelmingly HIV driven, with tuberculosis, latent or active, affecting chorismate/aromatic amino acid metabolism pathways (Figure 4).

### Fecal and plasma biomarkers differences in HIV co-infected individuals with latent and active tuberculosis

**Figure 5.**
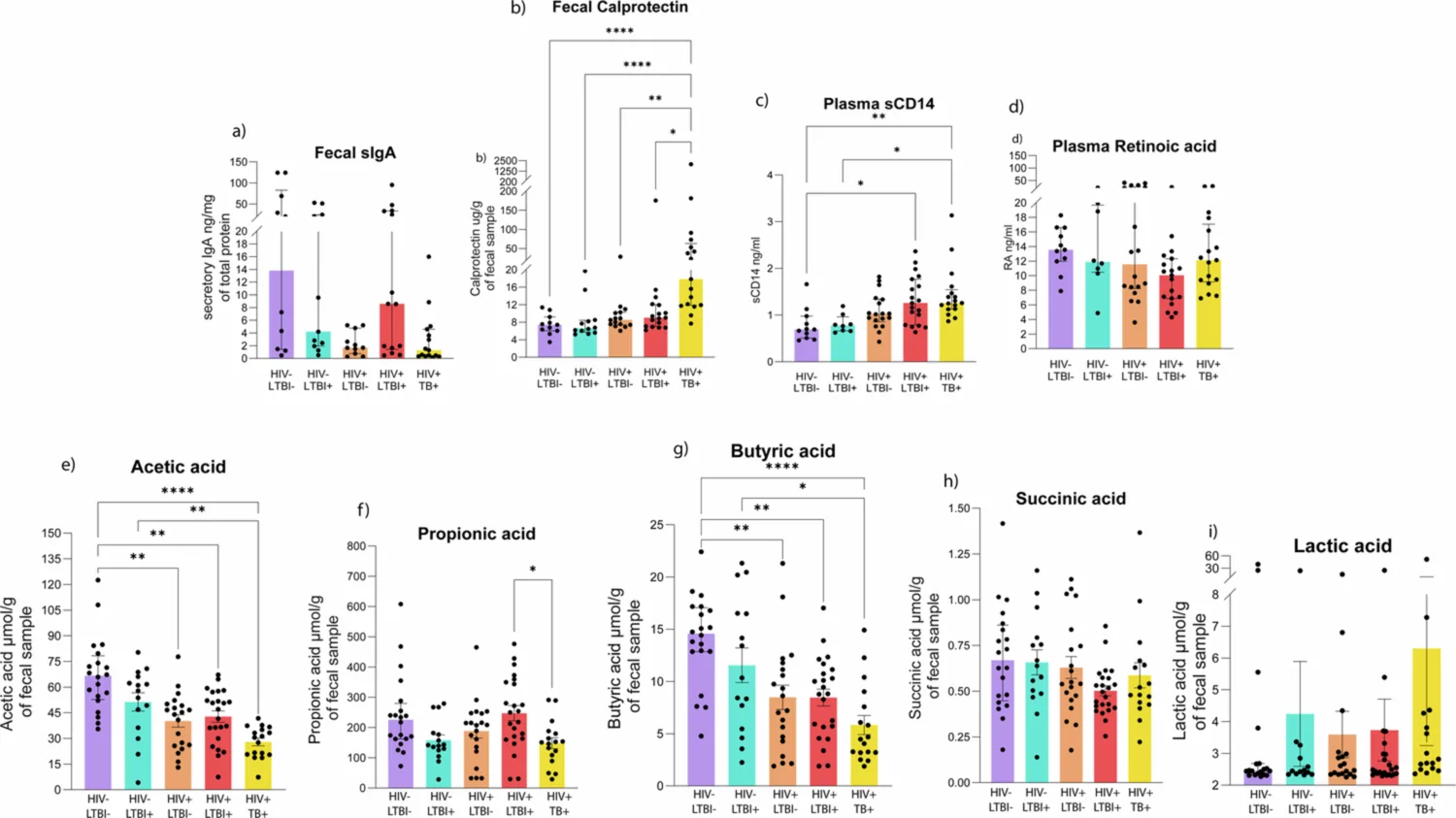
Estimation of fecal and plasma markers of microbial translocation, immune activation, fecal short-chain fatty acids (SCFAs). Levels of (a) fecal secretory IgA, (b) fecal calprotectin, (c) plasma soluble CD14 (sCD14), (d) plasma retinoic acid, (e) fecal acetic acid, (f) propionic acid, (g) butyric acid, (h) succinic acid, and (i) lactic acid were quantified in the study groups. Fecal secretory IgA was measured in HIV−LTBI− (*n* = 10), HIV−LTBI+ (*n* = 11), HIV+LTBI− (*n* = 11), HIV+LTBI+ (*n* = 14), and HIV+TB+ (*n* = 15). Fecal calprotectin was measured in HIV−LTBI− (*n* = 11), HIV−LTBI+ (*n* = 12), HIV+LTBI− (*n* = 15), HIV+LTBI+(*n* = 17), and HIV+TB+ (*n* = 17). Plasma sCD14 and retinoic acid were measured in HIV−LTBI− (*n* = 11), HIV−LTBI+ (*n* = 8), HIV+LTBI− (*n* = 18), HIV+LTBI+ (*n* = 19), and HIV+TB+ (*n* = 17). SCFAs were measured in HIV−LTBI− (*n* = 20), HIV−LTBI+ (*n* = 15), HIV+LTBI− (*n* = 20), HIV+LTBI+ (*n* = 23), and HIV+TB+ (*n* = 17). Fecal and plasma markers were quantified by ELISA, whereas SCFAs were quantified by HPLC. Data are presented as median with interquartile range (IQR). Statistical significance was determined using the Kruskal–Wallis test followed by Dunn's multiple-comparison post hoc test. *p < 0.05; **p < 0.01; ***p < 0.001; ****p < 0.0001.

In view of the importance of changes in the gut microbiome associated with immune responses locally and systemically, we compared immunological factors which are associated with the gut microbiome and HIV by performing ELISA. Secretory IgA (sIgA) is a key component of the first line of defense and elicits immunological response in the gut mucosal region. A trend towards increase in fecal sIgA levels was seen in the HIV+LTBI+ group whereas the HIV+LTBI− and HIV+TB+ groups had comparatively lower levels (Figure 5a). Calprotectin has immune-regulatory functions and fecal calprotectin is a marker of intestinal inflammation which was significantly different across our study groups. HIV+TB+ showed significantly increased fecal calprotectin level suggesting an altered immune-regulatory potential in these individuals (Figure 5b). Soluble CD14 (sCD14) is a marker of immune activation associated with microbial translocation and systemic inflammation. Blood plasma sCD14 levels were significantly different across groups, with significantly higher levels in HIV+LTBI+ and HIV+TB+ groups compared to other groups, with a trend showing steady increase in the median value with increase in disease severity (Figure 5c). Retinoic acid produced by dendritic cells helps in the gut homing of immune cells, which maintain gut mucosal immunity. Hence, retinoic acid levels were measured in plasma samples. The plasma retinoic acid levels differed across groups, with lower levels in the HIV+LTBI+ group, though the difference was not significant (Figure 5d).

### Fecal SCFA levels reduce with the disease severity

Fecal short-chain fatty acids (SCFAs), which are key microbial metabolites implicated in immune regulation, were quantified using HPLC. Figure 5(e–i) showed there was a significant decrease in SCFA levels with disease progression wherein acetic acid and butyric acid decreased compared to HIV-negative groups (Figure 5e and g). Propionic acid was significantly higher in HIV+LTBI+ group (247.3 ± 119.9 µmol/g) compared to HIV+TB+ (148.1 ± 74.55 µmol/g) (Figure 5f). Lactic acid was seen to have the opposite trend to that of the other SCFAs wherein it was higher in HIV+TB+ (6.30 ± 12.21 µmol/g), though the difference was not significant (Figure 5i). No significant difference in succinic acid was seen across the groups (Figure 5h). Progression to active TB is marked by microbial configurations linked to barrier dysfunction, microbial translocation, and heightened systemic immune activation.

### Sequential remodeling of microbiome–immune–metabolite associations across HIV and TB disease states

**Figure 6.**
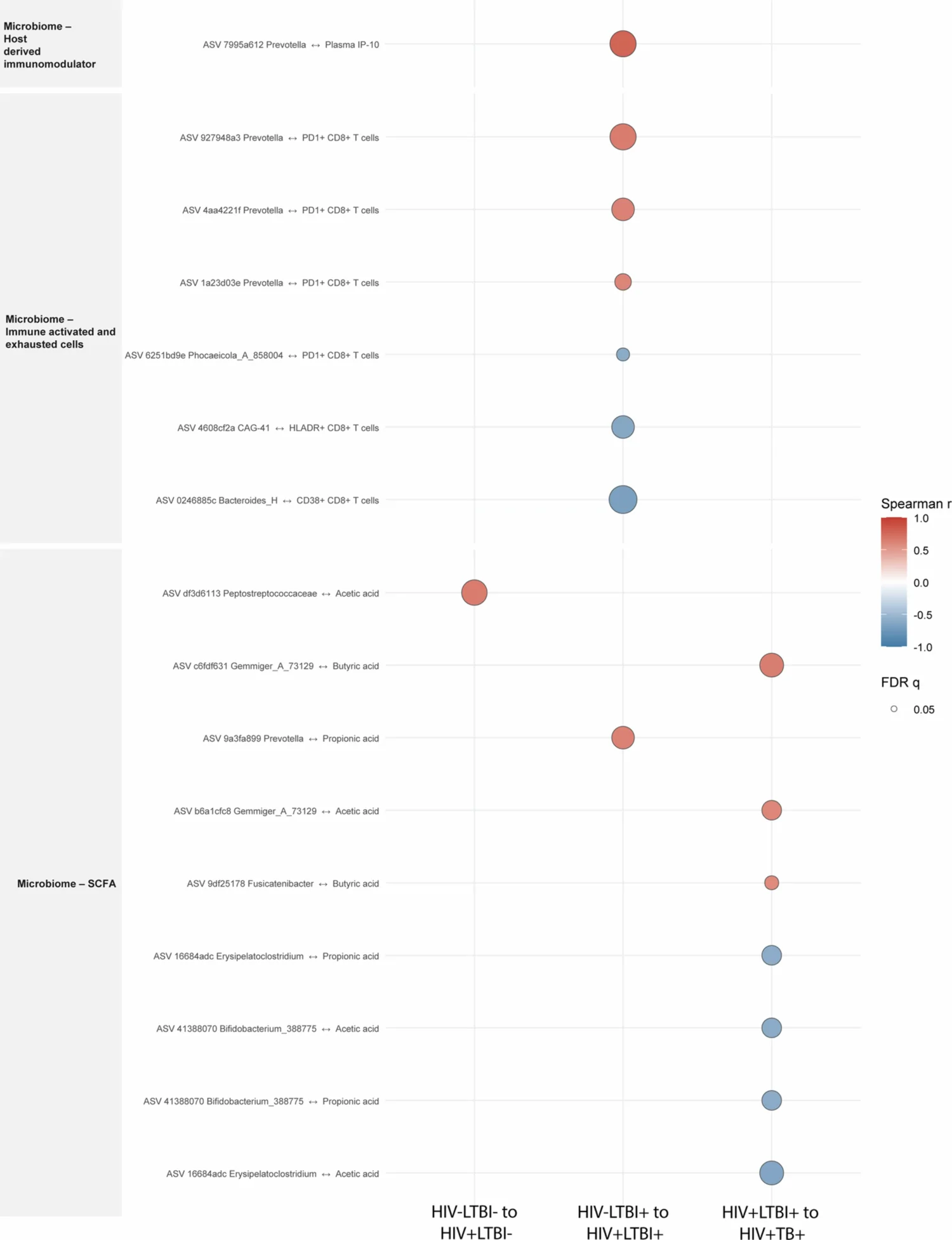
Transition of significant microbiome–host associations across disease states following FDR correction. Significant Spearman correlations (Benjamini–Hochberg false discovery rate [FDR] *q* < 0.05) between gut microbial amplicon sequence variants (ASVs) and host-derived immunomodulators, immune activation/exhaustion markers, and short-chain fatty acids (SCFAs) are shown across three pooled cohort transitions: HIV−LTBI− to HIV+LTBI−, HIV−LTBI+ to HIV+LTBI+, and HIV+LTBI+ to HIV+TB+. Each row represents a unique microbiome–host feature pair, grouped by interaction category (microbiome–host-derived immunomodulator, microbiome–immune activated/exhausted cells, and microbiome–SCFA). Circles are displayed only for statistically significant correlations within each pooled cohort. Circle color indicates the Spearman correlation coefficient (*r*), with red representing positive correlations and blue representing negative correlations. Circle size is proportional to statistical significance (smaller FDR *q*-values correspond to larger circles).

To characterize the dynamic remodeling of host–microbiome interactions during HIV and tuberculosis disease progression, correlation analyses were performed on pooled cohorts representing sequential disease states. Specifically, correlations were evaluated across three transitions: (i) HIV−LTBI− to HIV+LTBI− (ii) HIV−LTBI+ to HIV+LTBI+ (iii) HIV+LTBI+ to HIV+TB+. This pooled cohort approach enabled analysis within a broader biological state, revealing associations among microbiome ASVs, immune cell populations, SCFAs and immune mediators and how these associations change as disease progresses. Across the three transitions, 16 correlations were significant after FDR correction (*q* < 0.05) (Figure 6). In the HIV−LTBI− to HIV+LTBI− transition, only a single association survived correction, i.e. an unclassified Peptostreptococcaceae ASV correlated positively with acetic acid (*r* = +0.65, *q* = 0.024). This suggests that HIV infection alone, in the absence of LTBI, was associated with relatively minor reorganization of microbiome‒metabolite associations. In contrast, the HIV−LTBI+ transition to HIV+LTBI+ produced eight significant correlations, dominated by ASVs of *Prevotella* linked to CD8⁺ T-cell activation/exhaustion and interferon-driven inflammation. Three *Prevotella* ASVs correlated positively with PD1⁺ CD8⁺ T cells (*r* = +0.61; *q* = 0.023 − 0.040), and *Prevotella* also correlated positively with propionic acid (*r* = +0.62, *q* = 0.030) and, most strongly of all edges in the study, with plasma IP-10 (*r* = +0.78, *q* = 0.023). Reciprocally, *Bacteroides_H* was inversely associated with CD38⁺ CD8⁺ T cells (*r* = −0.68, *q* = 0.020), *CAG-41* with HLA-DR⁺ CD8⁺ T cells (*r* = −0.62, *q* = 0.030), and *Phocaeicola_A* with PD1⁺ CD8⁺ T cells (*r* = −0.60, *q* = 0.045). The activated/exhausted CD8 populations (PD1⁺, CD38⁺, and HLA-DR⁺) all increased in HIV infected individuals in the backdrop of LTBI (Supplementary Figure 7). ASV *Prevotella* increased while *Bacteroides_H*, *Phocaeicola_A*, and *CAG-41* decreased in HIV+LTBI+. In the transition to HIV+TB+ from HIV+LTBI+, all seven significant associations were microbiome-SCFA, and their content reflected a collapse of butyrogenic function. Butyrate producers such as *Gemmiger_A* and *Fusicatenibacter* correlated positively with butyric and acetic acid (*r* = +0.58–0.64), and both these producers and their SCFAs declined together in HIV+TB+. In parallel, ASVs of *Bifidobacterium* and *Erysipelatoclostridium* were both inversely correlated with acetic and propionic acid (*r* = −0.60 to −0.63). Notably, both taxa increased while SCFAs levels fell, indicating a functional uncoupling of these taxa from fermentative output. Therefore, these findings reveal a stepwise reorganization of host‒microbiome interactions, wherein it was minimal in the presence of HIV alone, inflammatory and *Prevotella*-dominated with LTBI in the background and finally coordinated the loss of butyrate-producing bacteria and their anti-inflammatory metabolites, which mirrors the immunometabolic deterioration accompanying disease progression.

### ART induces early microbiome remodeling followed by stabilization

**Figure 7.**
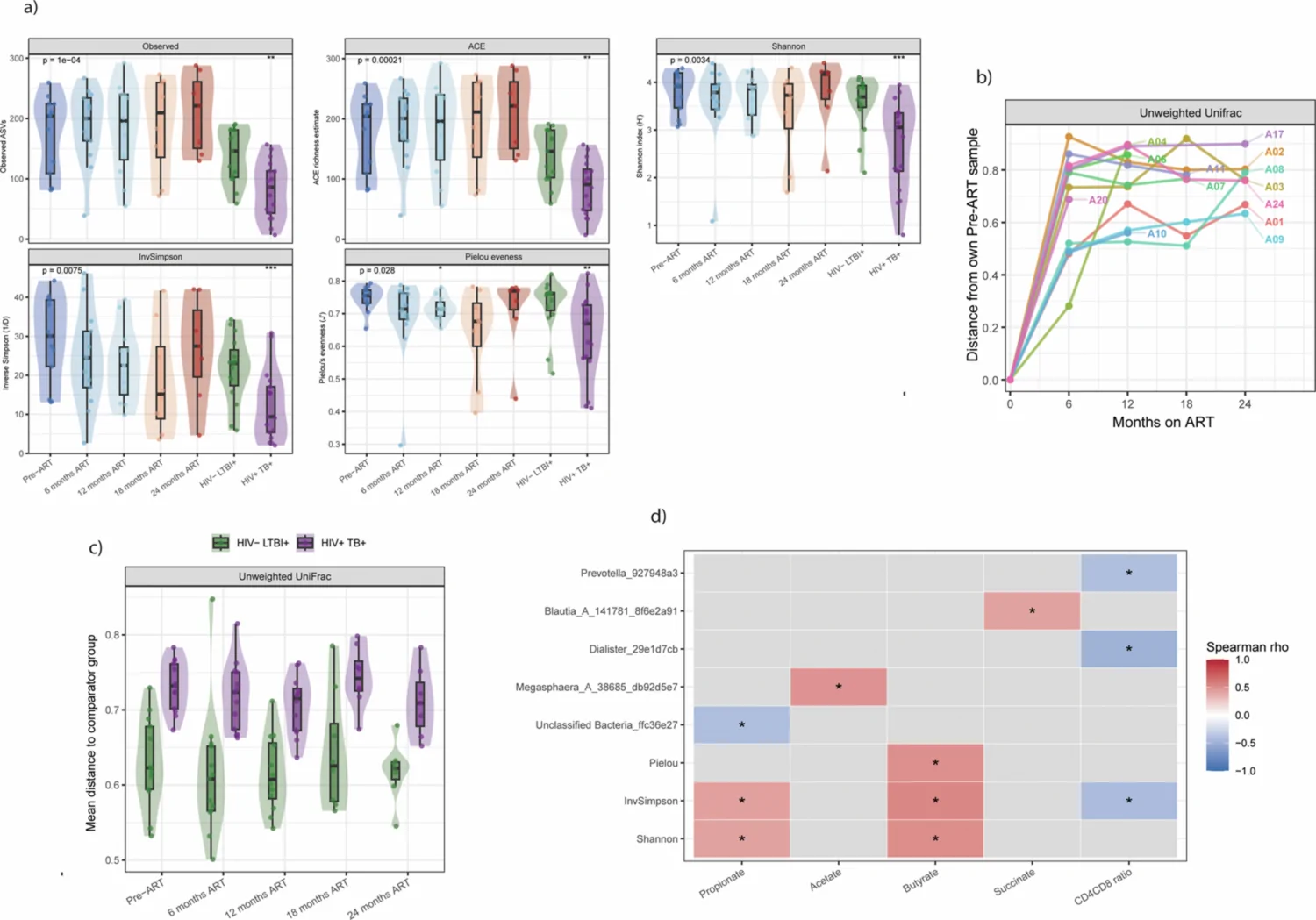
Longitudinal changes in the gut microbiome following ART initiation in HIV+LTBI+ participants. (a) Alpha diversity (observed ASVs, ACE, Shannon, inverse Simpson, and Pielou's evenness) across pre-ART, 6-, 12-, 18-, and 24-month ART time points compared with HIV−LTBI+ and HIV+TB+ cross-sectional comparator groups. Global differences were assessed by Kruskal–Wallis test with pairwise Wilcoxon tests versus pre-ART. (b) Within-participant microbiome divergence from the pre-ART baseline measured using unweighted UniFrac distance over 24 months of ART. (c) Mean unweighted UniFrac distance of the on-ART samples to the HIV−LTBI+ and HIV+TB+ comparator groups, where lower values indicate greater similarity. (d) Significant Spearman correlations (Benjamini–Hochberg adjusted *p* < 0.05) between microbiome features (alpha diversity indices and selected ASVs) and clinical/metabolic parameters, including fecal short-chain fatty acids and CD4/CD8 ratio. Sample sizes were pre-ART (*n* = 13), 6 months (*n* = 13), 12 months (*n* = 12), 18 months (*n* = 8), and 24 months (*n* = 7).

Over 24 months of ART, the gut community structure was broadly stable, with no ASV reached significance versus the pre-ART baseline by either ANCOM-BC2 or MaAsLin2. No alpha-diversity metric differed significantly from baseline at any timepoint (Figure 7a) (all BH-adjusted *p* ≥ 0.10; the strongest signal was a modest, non-significant dip in Pielou evenness at 18 months), Continuous-time models showed no significant temporal slope for any metric (all *p* > 0.18), with IPT and CPT contributing no detectable effect. Against the cross-sectional anchors, however, the treated cohort occupied a consistent position wherein alpha diversity was statistically indistinguishable from HIV−LTBI+ controls at essentially all timepoints, yet significantly higher than the HIV+TB+ group across observed, ACE, Shannon and Inverse Simpson diversity (Figure 7a) (multiple comparisons BH *p* < 0.05). Beta diversity distances mirrored this, with on-ART samples lying consistently closer to the HIV−LTBI+ group (median Bray‒Curtis = 0.72) than to the HIV+TB+ group (=0.82) at every time points and for UniFrac metrics (Figure 7c). Exploratory correlations linked microbial diversity to host and microbial metabolic markers. Butyrate correlated positively with Inverse Simpson, Shannon and evenness (*r* = 0.50 to 0.53, *q* = 0.01), propionate and acetate with diversity and with *Megasphaera*, and succinate with *Blautia* (all *q* < 0.05), whereas the CD4/CD8 ratio correlated inversely with diversity and with *Dialister* and *Prevotella* (r = −0.41 to −0.49, *q* = 0.01–0.05) (Figure 7d), suggesting that immune recovery and microbial metabolic output may not evolve synchronously during ART. No significant associations were detected with the viral load. Among 13 HIV+LTBI+ individuals who were followed up, 1 individual was reactivated for TB after 12 months on ART and CPT and also completing the course of IPT for 6 months. This isolated event has been reported descriptively and was not analyzed further because of the limited sample size.

## Discussion

The immune system maintains a fine balance of tolerance in the case of latent tuberculosis infection, wherein the immune response is just enough to keep the bacilli in check for most of the population.^19^ This delicate balance is disrupted owing to the presence of HIV, resulting in persistent chronic inflammation due to gut microbiome dysbiosis and a consequent increase in the translocation of microbial metabolites.^20^ As a result, individuals with LTBI are at substantially greater risk of progressing to active tuberculosis. We reasoned that, against the dysbiotic background that HIV imposes on the gut, latent TB infection (LTBI) is associated with a distinct and comparatively contained microbial–immune state, and that departure from this state, whether the loss of LTBI−associated features or progression to active disease would coincide with a breakdown of that containment. Our cross-sectional comparison across the HIV-TB spectrum, complemented by an exploratory longitudinal follow-up of HIV+LTBI+ individuals, was designed to test whether the microbiome tracks these clinical states. Previous gut microbiome studies in the patient groups, similar to the present study, are limited by small sample sizes and cross-sectional study design^21^ ^,^ ^22^ which highlights a critical gap that our integrated cross-sectional and longitudinal analyses aimed to address. As the study is observational, we present these findings as associations that generate testable hypotheses about pathogenesis rather than as evidence of causation.

The prevailing view treats low within-sample diversity as a hallmark of a diseased, less resilient gut and higher diversity as a correlate of stability and resistance to perturbation.^23^ From this premise, one would predict diversity to fall steadily across our severity gradient. In contrast, alpha diversity in HIV+LTBI+ was comparable to that of HIV-negative individuals and, by the inverse Simpson index, similar to or higher than in the other HIV-positive groups, with the clear loss of richness and rise in single taxon dominance largely confined to HIV+TB+ group. Consistent with the concept that LTBI represents a distinct biological state, our recent immune profiling study showed that PLHIV with LTBI exhibit distinct immune signatures characterized by reduced T-cell activation and PD-1 expression.^8^ Together, these findings suggest that latent TB infection in PLHIV is characterized by coordinated differences across both the gut microbiome and the immune system, although the mechanistic relationship between these features remains to be determined. This finding also helps explain why studies comparing HIV-positive and HIV-negative groups have reached inconsistent conclusions about diversity.^24^ If LTBI status is not measured, it could partly account for this discrepancy. Beta diversity reinforced this picture that HIV+LTBI+ formed the most distinct community in the cohort, separating from both HIV+LTBI− and HIV+TB+, whereas among apparently healthy individuals, LTBI status alone did not shift community structure. We therefore propose that LTBI in the context of HIV is associated with a particular microbial equilibrium and that movement away from it coincides with community reorganization.

The compositional changes were consistent with this framework. Proteobacteria, which expand in several intestinal diseases,^25^ were enriched across all HIV-positive groups and appeared to be a signature of HIV-associated dysbiosis rather than of TB. The feature that most set HIV+LTBI+ apart was a marked increase in Bacteroidota at the expense of fermentative Firmicutes, giving this group the lowest Firmicutes-to-Bacteroidota (F/B) ratio in the cohort. A reduced F/B ratio has been linked to metabolic and viral disease states,^26^ ^,^ ^27^ and the accompanying increase in Bacteroidota is consistent with the higher fecal propionate levels measured in this group.^28^

These phylum-level shifts were recapitulated, and given clear directionality, at the level of individual ASVs. Against the HIV background, active TB carried a strongly unidirectional signature of the ASVs that separated HIV+TB+ from the other two HIV-positive groups, which included lactic-acid bacteria^29^ (*Lactobacillus*, *Ligilactobacillus*, *Limosilactobacillus*) together with several taxa usually regarded as butyric acid producers,^30^ such as *Roseburia*, *Faecalibacterium*, *Agathobaculum* and *Blautia* which are not themselves lactate utilisers.^31^ This observation is also reiterated by studies on gut microbiome of Indian population, wherein there is increased *Faecalibacterium*, *Roseburia*, *Eubacterium* and *Phascolarctobacterium* and reduced *Prevotella* and *Bifidobacterium* in TB infected individuals when compared to healthy controls.^32^ In the inflamed, lactate-accumulating environment of HIV+TB+, lactate generated by the co-enriched lactic-acid bacteria may not be efficiently converted to butyrate and propionate.^33^ This interpretation is directly supported by the ecology of colonic cross-feeding, in which primary fermenters and lactate/acetate producers supply substrate to specialist butyrogens, so that disruption of the interactions rather than simple loss of producer taxa collapses butyrate output.^34^ The HIV+LTBI+ signature relative to HIV+LTBI− was defined more by specific depletion than enrichment, with a coordinated loss of commensal Clostridia, including butyrate-producers^34^ like *Intestinibacter*, *Coprococcus*, *Gemmiger* and *Mediterraneibacter*. This loss gives an ASV-level account of the low F/B ratio and likely contributes to its separation as a distinct microbial community. Notably, despite the depletion of these taxa, fecal propionate concentrations were maintained, suggesting that alterations in microbial community composition were not accompanied by a uniform reduction in all SCFAs. Although some ASVs assigned to *Megasphaera*, *Prevotella*, *Bifidobacterium* and *Streptococcus* were higher in HIV+LTBI+, distinct *Prevotella* ASVs moved in opposite abundance patterns, arguing against treating the genus as a functionally uniform taxon. *Prevotella* strains span commensal, fiber-associated, and pro-inflammatory phenotypes^35^ and are also abundant in the Indian gut microbiome.^36^

A notable strength of this study is the opportunity to interpret *Prevotella* signatures in a predominantly heterosexual cohort of people living with HIV, in which only a single HIV-positive participant reported men who have sex with men (MSM) behaviour. This distinction is important because the intestinal *Prevotella* enrichment reported in many HIV cohorts has been shown to be driven largely by MSM status and associated sexual behaviour rather than by HIV infection itself.^37^ The near-absence of MSM behaviour in our cohort therefore reduces this well recognised source of confounding and indicates that the *Prevotella*-associated signatures observed in HIV+LTBI+ individuals are more likely to reflect HIV- and LTBI−related alterations than behaviourally driven differences in microbiome composition.

The PICRUSt2-predicted functional profiles further reinforced the functional distinction between the gut microbiomes associated with HIV and TB. HIV groups shared an expansion of pathways for respiratory-quinone, heme and iron-acquisition and Gram-negative envelope functions expected of the Proteobacterial expansion.^38^ ^,^ ^39^ The lowest predicted capacity for chorismate biosynthesis and L-tyrosine degradation in HIV+LTBI+ is consistent with the coordinated loss of commensal Clostridia, including *Intestinibacter*, *Coprococcus* which have been broadly shown to contribute to the broader community capacity for aromatic amino acid transformation in the gut.^40^ ^,^ ^41^ Because bacterial processing of aromatic amino acids yields circulating metabolites (e.g. phenolic and indolic derivatives, AhR ligands) that tune mucosal barrier integrity and favor Treg over Th17 skewing,^41^ a reduced community-level capacity to metabolize tyrosine may attenuate this immunomodulatory output at the mucosa. This parallels the tryptophan-catabolism dysregulation already linked to HIV mucosal immune dysfunction^20^ and suggests that a diminished aromatic-amino-acid metabolic potential could contribute to the permissive immune environment associated with TB containment. This also parallels with reduced CD4+ Tregs frequency in HIV+LTBI+ individuals compared to other HIV+LTBI−.^8^ As our functional inferences are based on PICRUSt2 predictions, this hypothesis should be tested using targeted metabolomic approaches in these settings.

Co-occurrence network analysis suggested that these compositional changes are accompanied by reorganization of the interactions between community members. Keystone taxa drive community composition and function.^42^ Therefore, reliance on a limited number of highly connected hub taxa may increase susceptibility to community reorganization if these central taxa are disrupted. The HIV+LTBI+ network was densely connected yet poorly compartmentalized, carried a high proportion of negative associations, and was anchored in part by a Proteobacterial pathobiont acting as a network hub. It was also the most vulnerable network in the cohort, since removing a few highly connected members caused it to fragment far more readily than in the other groups, whereas random loss of members degraded all networks similarly. Therefore, we describe that this community is metabolically distinctive but structurally fragile, since a modest perturbation, for example, antimicrobial exposure, could disproportionately collapse it.

Having established that HIV and TB imprint distinct compositional and network signatures on the gut community, we asked whether these were mirrored by the mucosal and systemic mediators that would link the microbiome to host immunity. The fecal and plasma immunomodulators traced a coherent mucosal-to-systemic gradient that shifted with disease stage. Secretory IgA, the dominant effector of humoral mucosal defense and itself a shaper of commensal community structure,^43^ trended highest in HIV+LTBI+ group and fell in HIV+TB+, consistent with the notion that the LTBI−associated state retains a degree of mucosal immune competence that is lost as disease becomes active.^44^ Fecal calprotectin is a neutrophil-derived S100A8/A9 complex whose luminal concentration scales with neutrophil transmigration across an inflamed epithelium,^45^ was selectively elevated in HIV+TB+, as the point at which neutrophilic intestinal inflammation emerges.^46^ Systemically, plasma sCD14 rose stepwise with severity as a monocyte-shed co-receptor for LPS, sCD14 is a validated readout of microbial translocation and monocyte activation that independently predicts HIV disease progression and mortality,^47^ so its graded increase places progressive barrier dysfunction and innate immune activation occurring alongside the observed compositional changes. Taken together, these mediators suggest that HIV + LTBI+ group is associated with higher mucosal humoral defense, limited epithelial inflammation, and increasing systemic microbial translocation, whereas HIV+TB+ is characterized by intestinal inflammation together with the highest levels of systemic translocation.

The SCFA profile provided a microbial metabolic correlate of this trajectory. The acetate and butyrate levels declined progressively across the severity gradient. Because butyrate is the principal energy source of colonocytes and a potent inducer of colonic Foxp3⁺ Treg and IL-10 responses through GPR109A signaling and histone-deacetylase inhibition,^48^ its loss may contribute to both impaired barrier integrity and reduced mucosal immune regulation during active disease. Propionate is elevated in HIV+LTBI+ compared to HIV+TB+ group, in keeping with the Bacteroidota expansion and the lowest F/B ratio that distinguish this group, reinforcing the picture of a metabolically distinctive community. The reciprocal accumulation of lactate in the HIV+TB+ group, together with the enrichment of lactic acid-producing bacteria, is consistent with impaired microbial cross-feeding.^31^

The staged correlation analysis integrated these observations into a directional narrative, where this analysis approximates the sequence an individual would traverse in a high-burden setting such as India, where LTBI is widespread and HIV co-infection markedly accelerates progression to active disease. The near-absence of significant edges at HIV acquisition, i.e. a single ASV Peptostreptococcaceae–acetate association, suggests that, against an LTBI−negative background, HIV alone reorganized microbiome-immune interactions only modestly. In contrast, the transition to HIV+LTBI+ was dominated by an interferon-inflammatory module in which multiple *Prevotella* ASVs correlated positively with activated/exhausted PD1⁺ CD8⁺ T cells and plasma IP-10. Intestinal *Prevotella* enrichment in HIV has been linked to dendritic-cell and CD8 T-cell activation and to dysregulated type-I-interferon signaling,^49^ while IP-10 (CXCL10) is an interferon-γ-induced chemokine that rises with both HIV progression and mycobacterial infection.^50^ *Bacteroides_H*, *Phocaeicola_A* and *CAG-41*, which decreased as *Prevotella* increased, correlated inversely with the activated CD8 compartments, identifying them in the lower-activation state that gives way as Prevotella expands. We hypothesize that this replacement of commensals by *Prevotella* in the apparently healthy HIV−LTBI+ group may mark as the switch into an activated, interferon-associated CD8 profile in HIV+LTBI+. The final transition from HIV+LTBI+ to HIV+TB+ carried only microbiome-SCFA associations, and all indicated a collapse of butyrogenic function. Canonical butyrate producers (*Gemmiger_A* and *Fusicatenibacter*) declined in step with butyrate and acetate, whereas *Bifidobacterium* and *Erysipelatoclostridium* became inversely coupled to acetate and propionate, increasing even as those metabolites fell. Because *Bifidobacterium* is chiefly a lactate and acetate producer that fuels butyrogenesis through cross-feeding rather than generating butyrate itself,^31^ this pattern is consistent with the disruption of cross-feeding interactions, resulting in reduced production of anti-inflammatory SCFAs during active disease.

Our exploratory longitudinal follow-up of HIV+LTBI+ individuals on ART offers a tentative window onto how this microbial–immune equilibrium behaves once treatment is introduced. Rather than converging toward the diversity and structure of HIV-negative controls, the treated community showed early change and then stabilized at a configuration that remained statistically indistinguishable from cross-sectional HIV-LTBI+ controls and consistently distinct from the HIV+TB+ configuration, both in alpha diversity and UniFrac distances. The exploratory correlations are congruent with this reading, with butyrate and other SCFAs tracking positively with diversity and the CD4/CD8 ratio tracking inversely, this suggests that microbial diversity recovery may be temporally uncoupled from the numeric immune reconstitution in terms of the numbers of CD4 and CD8 cells following ART.

## Limitations and conclusions

Several limitations should be considered when interpreting these findings. First, this was an observational study, and therefore all findings should be interpreted as associations rather than evidence of causal relationships. Although the primary analyses were adjusted for age, sex, BMI, and diet, residual confounding by incompletely measured or unmeasured factors, including socioeconomic and behavioral variables, cannot be excluded. Second, the longitudinal cohort was small and subject to attrition (13 participants at enrolment and 7 at the final follow-up), with only a single TB reactivation event. Consequently, the longitudinal analyses were underpowered, and observations relating to microbiome changes following ART, immune restoration, SCFA dynamics, and TB reactivation should be regarded as exploratory and hypothesis-generating. Third, the absence of an HIV-negative active TB comparator limits our ability to distinguish microbiome alterations attributable to active TB alone from those associated with HIV–TB coinfection. Finally, taxonomic and functional interpretations are constrained by the use of 16S rRNA gene sequencing and PICRUSt2. Differential abundance was therefore interpreted primarily at the ASV/genus level, and predicted functional pathways should not be considered direct measurements of microbial activity. Validation using shotgun metagenomics, metatranscriptomics, targeted metabolomics, and larger longitudinal cohorts will be important to confirm and extend these findings.

Despite these limitations, our integrated cross-sectional and exploratory longitudinal analyses consistently identify LTBI as being associated with a distinct gut microbiome configuration and immune-metabolic profile in people living with HIV. These findings suggest that microbiome alterations accompanying LTBI differ from those observed in active TB and highlight potential host–microbiome interactions that merit further investigation. Future studies incorporating larger longitudinal cohorts and multi-omics approaches will be essential to determine whether these microbial and immune-metabolic features are associated with progression from latent to active tuberculosis and to evaluate their potential as biomarkers of disease progression.

## Supplementary Material

Supplementary table 3.docx

Supplementary material Supplementary table 2.docx

Supplementary material Supplementary table 1.docx

Supplementary material Supplementary table 4.docx

Supplementary material KGMI_A_2721739_SM2728.tif

Supplementary material KGMI_A_2721739_SM2727.tif

Supplementary material KGMI_A_2721739_SM2725.tif

Supplementary material KGMI_A_2721739_SM2723.tif

Supplementary material KGMI_A_2721739_SM2721.tif

Supplementary material KGMI_A_2721739_SM2720.tif

## Data Availability

The raw sequencing data have been deposited in the NCBI Sequence Read Archive (SRA). The bioproject ID is PRJNA1428159.
