# Supplementary material for: Latent tuberculosis infection shapes gut microbiome and immune-metabolic interactions in people living with HIV
Source: Gut Microbes. 2026 Aug 27;18(1):2721739. doi: 10.1080/19490976.2026.2721739 (PMC13524005; doi:10.1080/19490976.2026.2721739)
Supplement: Supplementary file 1 [file KGMI_A_2721739_SM2722.docx]

| **Parameter** | **HIV-LTBI-** | **HIV-LTBI+** | **HIV+LTBI-** | **HIV+LTBI+** | **HIV+TB+** |
| --- | --- | --- | --- | --- | --- |
| Nodes (taxa) | 138 | 124 | 179 | 166 | 167 |
| Edges | 287 | 429 | 1390 | 553 | 433 |
| Positive edges | 275 | 362 | 1353 | 503 | 423 |
| Negative edges | 12 | 67 | 37 | 50 | 10 |
| Average degree | 4.16 | 6.92 | 15.53 | 6.66 | 5.19 |
| Connectance (density) | 0.03 | 0.056 | 0.087 | 0.04 | 0.031 |
| Clustering coefficient | 0.38 | 0.45 | 0.47 | 0.31 | 0.44 |
| Network modules | 12 | 11 | 5 | 4 | 6 |
| Relative modularity | 0.28 | −0.07 | 0.69 | 0.26 | 0.71 |
| Average path length | 2.49 | 3.06 | 1.77 | 2.55 | 3.39 |
| Keystone taxa | 5 | 11 | 10 | 21 | 4 |
| Node loss at 6th hub removed (targeted, %) | 17.7 | 29.8 | 11.5 | 18.6 | 9.7 |
| Vulnerability | 0.088 | 0.146 | 0.015 | 0.087 | 0.079 |

**Supplementary Table 2:** Summary of co-occurrence network topology and robustness

metrices of study groups; HIV-LTBI- (n=20), HIV-LTBI+ (n=15), HIV+LTBI- (n=20),

HIV+LTBI+ (n=23) and HIV+TB+ (n=17).
