# Supplementary material for: Latent tuberculosis infection shapes gut microbiome and immune-metabolic interactions in people living with HIV
Source: Gut Microbes. 2026 Aug 27;18(1):2721739. doi: 10.1080/19490976.2026.2721739 (PMC13524005; doi:10.1080/19490976.2026.2721739)
Supplement: Supplementary file 2 [file KGMI_A_2721739_SM2724.docx]

| **SampleID** | **Group** | **n_ASVs** | **library_size** |  | **SampleID** | **Group** | **n_ASVs** | **library_size** |
| --- | --- | --- | --- | --- | --- | --- | --- | --- |
| A1 | HIV+LTBI+ | 428 | 348490 |  | C11 | HIV+TB+ | 286 | 415580 |
| A10 | HIV+LTBI+ | 407 | 226780 |  | C12 | HIV+TB+ | 307 | 440540 |
| A11 | HIV+LTBI+ | 247 | 253905 |  | C13 | HIV+TB+ | 373 | 494794 |
| A12 | HIV+LTBI+ | 130 | 525012 |  | C14 | HIV+TB+ | 181 | 335332 |
| A13 | HIV+LTBI+ | 401 | 459629 |  | C15 | HIV+TB+ | 468 | 526277 |
| A14 | HIV+LTBI+ | 338 | 464207 |  | C2 | HIV+TB+ | 92 | 283600 |
| A15 | HIV+LTBI+ | 414 | 579266 |  | C3 | HIV+TB+ | 105 | 273678 |
| A16 | HIV+LTBI+ | 293 | 472481 |  | C4 | HIV+TB+ | 296 | 293478 |
| A17 | HIV+LTBI+ | 234 | 457020 |  | C5 | HIV+TB+ | 260 | 321417 |
| A18 | HIV+LTBI+ | 512 | 492095 |  | C6 | HIV+TB+ | 40 | 352912 |
| A19 | HIV+LTBI+ | 265 | 476768 |  | C7 | HIV+TB+ | 216 | 384488 |
| A2 | HIV+LTBI+ | 306 | 314572 |  | C8 | HIV+TB+ | 207 | 352094 |
| A20 | HIV+LTBI+ | 615 | 360213 |  | C9 | HIV+TB+ | 96 | 388384 |
| A21 | HIV+LTBI+ | 366 | 354262 |  | D1 | HIV-LTBI+ | 384 | 331410 |
| A22 | HIV+LTBI+ | 151 | 302460 |  | D10 | HIV-LTBI+ | 573 | 460804 |
| A23 | HIV+LTBI+ | 377 | 487235 |  | D11 | HIV-LTBI+ | 402 | 509164 |
| A3 | HIV+LTBI+ | 392 | 317704 |  | D12 | HIV-LTBI+ | 545 | 459111 |
| A4 | HIV+LTBI+ | 408 | 360348 |  | D13 | HIV-LTBI+ | 509 | 475313 |
| A5 | HIV+LTBI+ | 507 | 271414 |  | D14 | HIV-LTBI+ | 551 | 442574 |
| A6 | HIV+LTBI+ | 235 | 283688 |  | D15 | HIV-LTBI+ | 353 | 470985 |
| A7 | HIV+LTBI+ | 197 | 267735 |  | D2 | HIV-LTBI+ | 251 | 283494 |
| A8 | HIV+LTBI+ | 411 | 281758 |  | D3 | HIV-LTBI+ | 188 | 330349 |
| A9 | HIV+LTBI+ | 429 | 264258 |  | D4 | HIV-LTBI+ | 230 | 225421 |
| B1 | HIV+LTBI- | 111 | 260398 |  | D5 | HIV-LTBI+ | 758 | 353445 |
| B10 | HIV+LTBI- | 113 | 260575 |  | D6 | HIV-LTBI+ | 100 | 255200 |
| B11 | HIV+TB+ | 101 | 358721 |  | D7 | HIV-LTBI+ | 272 | 361405 |
| B12 | HIV+LTBI- | 311 | 365192 |  | D8 | HIV-LTBI+ | 203 | 294910 |
| B13 | HIV+LTBI- | 313 | 392685 |  | D9 | HIV-LTBI+ | 862 | 503374 |
| B14 | HIV+LTBI- | 352 | 407205 |  | E1 | HIV-LTBI- | 198 | 428491 |
| B15 | HIV+LTBI- | 547 | 475646 |  | E10 | HIV-LTBI- | 355 | 354427 |
| B16 | HIV+LTBI- | 235 | 248287 |  | E11 | HIV-LTBI- | 286 | 327716 |
| B17 | HIV+LTBI- | 178 | 462465 |  | E12 | HIV-LTBI- | 584 | 392566 |
| B18 | HIV+LTBI- | 515 | 489201 |  | E13 | HIV-LTBI- | 348 | 471912 |
| B19 | HIV+LTBI- | 353 | 457295 |  | E14 | HIV-LTBI- | 165 | 337432 |
| B2 | HIV+LTBI- | 499 | 335506 |  | E15 | HIV-LTBI- | 414 | 453077 |
| B20 | HIV+LTBI- | 368 | 616946 |  | E16 | HIV-LTBI- | 286 | 501219 |
| B21 | HIV+LTBI- | 234 | 400381 |  | E17 | HIV-LTBI- | 350 | 435008 |
| B22 | HIV+LTBI- | 603 | 467927 |  | E18 | HIV-LTBI- | 271 | 501960 |
| B3 | HIV+TB+ | 315 | 268112 |  | E19 | HIV-LTBI- | 411 | 546912 |
| B4 | HIV+LTBI- | 247 | 267351 |  | E2 | HIV-LTBI- | 321 | 369046 |
| B5 | HIV+LTBI- | 101 | 309899 |  | E20 | HIV-LTBI- | 495 | 567900 |
| B6 | HIV+LTBI- | 67 | 215947 |  | E3 | HIV-LTBI- | 437 | 298215 |
| B7 | HIV+LTBI- | 344 | 297005 |  | E4 | HIV-LTBI- | 142 | 262625 |
| B8 | HIV+LTBI- | 103 | 249983 |  | E5 | HIV-LTBI- | 503 | 378502 |
| B9 | HIV+LTBI- | 260 | 359101 |  | E6 | HIV-LTBI- | 363 | 461654 |
| C1 | HIV+TB+ | 345 | 266034 |  | E7 | HIV-LTBI- | 496 | 343162 |
| C10 | HIV+TB+ | 197 | 383212 |  | E8 | HIV-LTBI- | 478 | 327430 |
|  |  |  |  |  | E9 | HIV-LTBI- | 487 | 471278 |

**Supplementary Table 1.** Sample metadata and sequencing statistics showing SampleID,

library size, number of ASVs, and study group assignment.
