# Supplementary material for: Latent tuberculosis infection shapes gut microbiome and immune-metabolic interactions in people living with HIV
Source: Gut Microbes. 2026 Aug 27;18(1):2721739. doi: 10.1080/19490976.2026.2721739 (PMC13524005; doi:10.1080/19490976.2026.2721739)
Supplement: Supplementary file 3 [file KGMI_A_2721739_SM2726.docx]

| **subject** | **Study_group** | **ART** | **IPT** | **CPT** | **CD4 count** | **CD4CD8 ratio** | **Viral load** |
| --- | --- | --- | --- | --- | --- | --- | --- |
| A01 | Pre-ART | Not on medication | Not on medication | Not on medication | 454.00 | 0.48 | 200347 |
| A01 | 6 months ART | On medication | On medication | Not on medication | 1421.23 | 0.95 | 32 |
| A01 | 12 months ART | On medication | Not on medication | Not on medication | 955.58 | 1.23 |  |
| A01 | 18 months ART | On medication | Not on medication | Not on medication | 293.17 | 1.44 |  |
| A01 | 24 months ART | On medication | Not on medication | Not on medication | 910.08 | 1.48 | 459 |
| A02 | Pre-ART | Not on medication | Not on medication | Not on medication | 409.00 | 0.39 | 562529 |
| A02 | 6 months ART | On medication | On medication | Not on medication | 526.05 | 1.16 |  |
| A02 | 12 months ART | On medication | Not on medication | Not on medication | 524.61 | 1.39 | 77 |
| A02 | 18 months ART | On medication | Not on medication | Not on medication | 465.97 | 1.61 | 3582 |
| A02 | 24 months ART | On medication | Not on medication | Not on medication | 747.92 | 1.56 |  |
| A03 | Pre-ART | Not on medication | Not on medication | Not on medication | 72.00 | 0.14 | 621950 |
| A03 | 6 months ART | On medication | Not on medication | On medication | 221.33 | 0.24 |  |
| A03 | 12 months ART | On medication | Not on medication | On medication | 296.58 | 0.40 | 199 |
| A03 | 18 months ART | On medication | Not on medication | On medication | 286.56 | 0.59 | 153 |
| A03 | 24 months ART | On medication | Not on medication | On medication | 308.56 | 0.65 | 450 |
| A04 | Pre-ART | Not on medication | Not on medication | Not on medication | 481.00 | 0.70 | 28031 |
| A04 | 6 months ART | On medication | On medication | Not on medication | 521.50 | 1.07 | 135 |
| A04 | 12 months ART | On medication | Not on medication | Not on medication | 588.00 | 0.90 | 202 |
| A06 | Pre-ART | Not on medication | Not on medication | Not on medication | 836.00 | 0.68 |  |
| A06 | 6 months ART | On medication | On medication | Not on medication | 1037.18 | 0.82 | 1233 |
| A06 | 12 months ART | On medication | Not on medication | Not on medication | 827.67 | 0.79 | 7148 |
| A07 | Pre-ART | Not on medication | Not on medication | Not on medication | 607.00 | 1.56 | 5621 |
| A07 | 6 months ART | On medication | On medication | Not on medication | 733.50 | 2.06 | 102 |
| A07 | 12 months ART | On medication | Not on medication | Not on medication | 524.10 | 1.85 | 323 |
| A07 | 18 months ART | On medication | Not on medication | Not on medication | 845.75 | 1.92 | 32 |
| A08 | Pre-ART | Not on medication | Not on medication | Not on medication | 381.00 | 0.35 | 63403 |
| A08 | 6 months ART | On medication | On medication | Not on medication | 494.35 | 0.59 | 1440 |
| A08 | 12 months ART | On medication | Not on medication | Not on medication | 656.10 | 0.63 | 2915 |
| A08 | 18 months ART | On medication | Not on medication | Not on medication | 611.61 | 0.75 | 1441 |
| A08 | 24 months ART | On medication | Not on medication | Not on medication | 786.17 | 1.06 | 1441 |
| A09 | Pre-ART | Not on medication | Not on medication | Not on medication | 343.00 | 0.39 | 217655 |
| A09 | 6 months ART | On medication | On medication | Not on medication | 863.40 | 0.58 | 79 |
| A09 | 12 months ART | On medication | Not on medication | Not on medication | 539.73 | 0.55 | 257 |
| A09 | 18 months ART | On medication | Not on medication | Not on medication | 723.99 | 0.71 | 134 |
| A09 | 24 months ART | On medication | Not on medication | Not on medication | 563.90 | 0.71 | 733 |
| A10 | Pre-ART | Not on medication | Not on medication | Not on medication | 369.00 | 0.44 | 28633 |
| A10 | 6 months ART | On medication | On medication | Not on medication | 607.79 | 0.96 | 185 |
| A10 | 12 months ART | On medication | Not on medication | Not on medication |  |  | 498 |
| A11 | Pre-ART | Not on medication | Not on medication | Not on medication | 468.00 | 0.20 | 75238 |
| A11 | 6 months ART | On medication | Not on medication | On medication | 413.91 | 0.35 |  |
| A11 | 12 months ART | On medication | Not on medication | On medication |  |  | 177 |
| A11 | 18 months ART | On medication | Not on medication | On medication | 616.42 | 0.65 | 423 |
| A17 | Pre-ART | Not on medication | Not on medication | Not on medication | 213.00 | 0.46 | 654 |
| A17 | 6 months ART | On medication | On medication | On medication | 263.39 | 0.22 | 7 |
| A17 | 12 months ART | On medication | Not on medication | On medication | 320.59 | 0.43 |  |
| A17 | 24 months ART | On medication | Not on medication | On medication | 422.91 | 0.48 | 19 |
| A20 | Pre-ART | Not on medication | Not on medication | Not on medication | 653.00 | 0.23 | 996 |
| A20 | 6 months ART | On medication | On medication | On medication | 914.19 | 0.57 | 244 |
| A23 | Pre-ART | Not on medication | Not on medication | Not on medication | 486.00 | 0.60 | 63530 |
| A23 | 6 months ART | On medication | On medication | Not on medication | 890.72 | 0.80 | 358 |
| A23 | 12 months ART | On medication | Not on medication | Not on medication | 843.65 | 1.10 | 2.E+12 |
| A23 | 18 months ART | On medication | Not on medication | Not on medication | 833.23 | 0.82 | 14436 |
| A23 | 24 months ART | On medication | Not on medication | Not on medication | 955.73 | 1.04 |  |

**Supplementary Table 4. Clinical, immunological, and treatment characteristics of study participants across ART follow-up time points.**

The table presents participant-level data for subjects enrolled in the study at different antiretroviral therapy (ART) follow-up periods: Pre-ART (before initiation of ART), and 6,12, 18, and 24 months after ART initiation. Variables include Subject ID, Study Group, ART duration, ART status (On medication/Not on medication), Isoniazid Preventive Therapy (IPT) status, Cotrimoxazole Preventive Therapy (CPT) status, CD4 count (cells/μL), CD4:CD8 ratio, and plasma viral load (copies/mL). "On medication" indicates that the participant was receiving the specified treatment at the time of sample collection, while "Not on medication" indicates that the treatment was not being received.
