# Supplementary material for: Latent tuberculosis infection shapes gut microbiome and immune-metabolic interactions in people living with HIV
Source: Gut Microbes. 2026 Aug 27;18(1):2721739. doi: 10.1080/19490976.2026.2721739 (PMC13524005; doi:10.1080/19490976.2026.2721739)
Supplement: Supplementary file 4 [file KGMI_A_2721739_SM2729.docx]

| **Groups** | | **Pre-ART** | **6M** | **12M** | **18M** | **24M** |
| --- | --- | --- | --- | --- | --- | --- |
| **Number of Subjects (n)** | | 13 | 13 | 12 | 8 | 7 |
| **Gender (females,males)** | | 3F, 10M |  |  |  |  |
| **CD4+ T cells (cells/µL)** | | 454  (72-836) | 608  (221-1421) | 564  (297-956) | 614  (287-846) | 748  (309-956) |
| **CD4/CD8 ratio** | | 0.44  (0.14-1.56) | 0.8  (0.22-2.06) | 0.85  (0.39-1.85) | 0.78  (0.59-1.93) | 1.04  (0.48-1.56) |
| **HIV-1 viral load (copies/mL)** | | 6.34 x 10^4^  (0-6.22 x 10^5^) | 1.02 x 10^2^  (0-1.44 x 10^3^) | 2.29 x 10^2^  (0-1.8 x 10^12^) | 2.88 x 10^2^  (0-1.44 x 10^4^) | 4.50x10^2^  (0-1.44X10^3^) |
| **Months on ART** | | 0 | 6 | 12 | 18 | 24 |
| **On Anti-Retroviral Therapy (n)** | Tenofovir (TDF) + Lamivudine (3TC) + Dolutegravir (DTG) | 0 | 13 | 12 | 7 | 7 |
|  | Abacavir (ABC) + Lamivudine (3TC) + Dolutegravir (DTG) | 0 | 0 | 0 | 1 | 0 |
| **On IPT for 6 months after 3 months of ART initiation (n)** | | 0 | 11 | 0 | 0 | 0 |
| **On CPT Cotrimoxazole Prophylactic Therapy (n)** | | 0 | 5 | 5 | 5 | 5 |
| **On Anti Tuberculosis Therapy (n)** | | 0 | 0 | 0 | 0 | 1 |
| **Adherence** | | 100% | 100% | 100% | 100% | 100% |

**Supplementary Table 3:** Ancilliary data of HIV+LTBI+ participants who were followed up

longitudinally. Data represented as median with range.
